# Iron-molybdenum cofactor synthesis by a thermophilic nitrogenase devoid of the scaffold NifEN

**DOI:** 10.1073/pnas.2406198121

**Published:** 2024-11-06

**Authors:** Lucía Payá-Tormo, Carlos Echavarri-Erasun, Natalia Makarovsky-Saavedra, Ana Pérez-González, Zhi-Yong Yang, Yisong Guo, Lance C. Seefeldt, Luis M. Rubio

**Affiliations:** ^a^Centro de Biotecnología y Genómica de Plantas, Universidad Politécnica de Madrid e Instituto Nacional de Investigación y Tecnología Agraria y Alimentaria/Consejo Superior de Investigaciones Científicas, Madrid 28223, Spain; ^b^Departamento de Biotecnología-Biología Vegetal, Escuela Técnica Superior de Ingeniería Agronómica, Alimentaria y de Biosistemas, Universidad Politécnica de Madrid, Madrid 28040, Spain; ^c^Department of Chemistry and Biochemistry, Utah State University, Logan, UT 84322; ^d^Department of Chemistry, Carnegie Mellon University, Pittsburgh, PA 15213

**Keywords:** nitrogen fixation evolution, metalloproteins, NifEN, *Roseiflexus*, FeMo-co

## Abstract

The maturation and installation of the active site metal cluster (FeMo-co, Fe_7_S_9_CMo-*R*-homocitrate) in Mo-dependent nitrogenase requires the protein product of the *nifB* gene for production of the FeS cluster precursor (NifB-co, [Fe_8_S_9_C]) and the action of the maturase complex composed of the protein products from the *nifE* and *nifN* genes. However, some putative diazotrophic bacteria, like *Roseiflexus* sp. RS-1, lack the *nifEN* genes, suggesting an alternative pathway for maturation of FeMo-co that does not require NifEN. In this study, the *Roseiflexus* NifH, NifB, and apo-NifDK proteins produced in *Escherichia coli* are shown to be sufficient for FeMo-co maturation and insertion into the NifDK protein to achieve active nitrogenase. The *E. coli* expressed NifDK*^RS^* contained P-clusters but was devoid of FeMo-co (referred to as apo-NifDK*^RS^*). Apo-NifDK*^RS^* could be activated for N_2_ reduction by addition of preformed FeMo-co. Further, it was found that apo-NifDK*^RS^* plus *E. coli* produced NifB*^RS^* and NifH*^RS^* were sufficient to yield active NifDK*^RS^* when incubated with the necessary substrates (homocitrate, molybdate, and *S*-adenosylmethionine [SAM]), demonstrating that these proteins can replace the need for NifEN in maturation of Mo-nitrogenase. The *E. coli* produced NifH*^RS^* and NifB*^RS^* proteins were independently shown to be functional. The reconstituted NifDK*^RS^* demonstrated reduction of N_2_, protons, and acetylene in ratios observed for *Azotobacter *vinelandii** NifDK. These findings reveal a distinct NifEN-independent pathway for nitrogenase activation involving NifH*^RS^*, NifB*^RS^*, and apo-NifDK*^RS^*.

## Significance

It is accepted that the minimum gene set for nitrogen fixation includes *nifH*, *nifD*, and *nifK* for nitrogenase, and *nifE*, *nifN,* and *nifB* for FeMo-co biosynthesis. Conventionally, the NifDK homolog NifEN is essential for FeMo-co synthesis. However, this study shows that a thermophilic *Roseiflexus* bacterium can assemble FeMo-co without NifEN. In this bacterium, NifDK serves both as a catalyst for nitrogen fixation and as a maturase for its own cofactor. This suggests a simpler pathway for FeMo-co synthesis predating the current *nifDK* and *nifEN* genes. This finding could simplify genetic transfer of nitrogen fixation to crops, which could enable them to utilize atmospheric N_2_.

Nitrogenases are enzyme complexes responsible for the reduction of inert N_2_ to bioavailable NH_3_ (1, 2). They are exclusively found in a selective group of microorganisms of the Bacteria and Archaea domains called diazotrophs that can use N_2_ as sole source of nitrogen (3, 4). All nitrogenases are composed of a dinitrogenase and a dinitrogenase reductase that contain FeS clusters necessary for N_2_ reduction. There are three types of nitrogenases that are genetically distinct and differ in the metal composition of their active site cofactors. The most common nitrogenase contains Fe and Mo, while alternative nitrogenases contain either V and Fe or Fe-only cofactors (5). The dinitrogenase component of the Mo nitrogenase is a NifDK heterotetramer of α_2_β_2_ composition while the dinitrogenase reductase is a NifH homodimer (6). NifH contains a site for ATP binding and hydrolysis in each subunit and a [Fe_4_S_4_] cluster that connects both subunits (7). NifDK contains a [Fe_8_S_7_] P-cluster at the interface of each αβ half and a [Fe_7_S_9_MoC(*R*)-homocitrate] FeMo-co at the active site of each α subunit (8–10). Electron transfer occurs during the association of both components, with transfer of an electron from the [Fe_4_S_4_] cluster of NifH to FeMo-co via the P-cluster, followed by the ATP hydrolysis and subsequent complex dissociation (11).

In addition to the structural *nifH*, *nifD,* and *nifK* genes, diazotrophs carry a variable number of *nif* genes, most of them involved in the biosynthesis of the nitrogenase metallo-cofactors (12). This variability in *nif*-gene composition is related to their physiology, metabolism, and ecological niche (13, 14). Furthermore, some *nif* functions might be performed by housekeeping counterparts encoded in non-*nif* genes. Both the physiological diversity and frequent functional overlap hinders the determination of the precise genetic requirements for N_2_ fixation in a specific diazotroph. The proposed minimal genetic requirement for Mo nitrogenases is associated with two major stages: the biosynthesis of FeMo-co, involving the genes *nifB*, *nifH*, *nifE,* and *nifN*, and the catalytic module formed by the nitrogenase structural genes *nifH*, *nifD,* and *nifK* (3, 12).

**Fig. 1.**
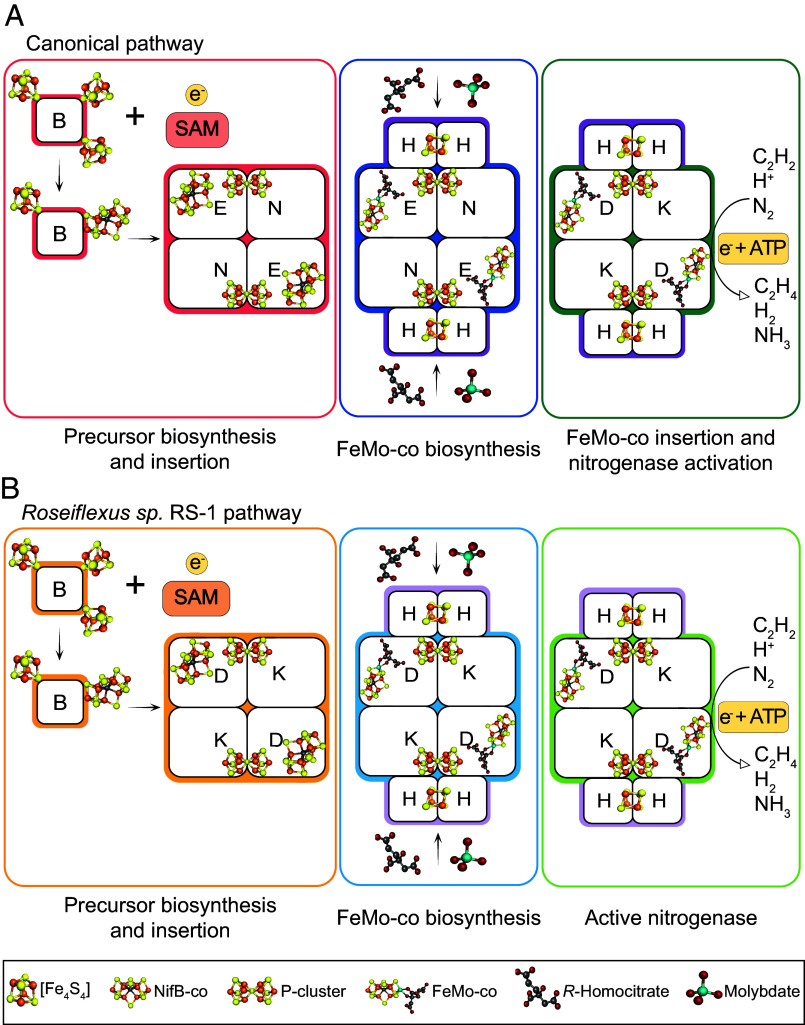
Schemes of FeMo-co biosynthetic pathways. (*A*) Canonical NifEN-dependent pathway as studied in *Azotobacter *vinelandii**. The *S*-adenosylmethionine [SAM]-radical enzyme NifB synthesizes the [Fe_8_S_9_C] cluster NifB-co, which is then inserted into NifEN. FeMo-co biosynthesis occurs at the NifEN scaffold by substituting Mo for an apical Fe atom and adding *R*-homocitrate. FeMo-co biosynthesis requires the interaction of NifH with NifEN. FeMo-co is then transferred to apo-NifDK (already containing mature P-clusters) to reconstitute active NifDK. (*B*) Putative NifEN-independent pathway of *Roseiflexus*. The SAM-radical enzyme NifB*^RS^* synthesizes the [Fe_8_S_9_C] cluster NifB-co, which is then inserted into apo-NifDK*^RS^* (already containing mature P-clusters or its precursors). FeMo-co biosynthesis occurs in situ in apo-NifDK*^RS^* and requires the interaction with NifH*^RS^*. After FeMo-co biosynthesis, mature NifDK*^RS^* is formed.

The biosynthesis of FeMo-co occurs outside of the NifDK protein. The first committed step in FeMo-co biosynthesis is the formation by NifB of a [Fe_8_S_9_C] cluster, referred to as NifB-co (15, 16), which is then transferred to the maturase complex, NifEN (17). Within NifEN and with the assistance of NifH, the precursor NifB-co is transformed into FeMo-co by the sequential addition of Mo (replacing an apical Fe atom) and homocitrate (ligated to the Mo atom) (18). Newly synthesized FeMo-co is finally transferred from NifEN to apo-NifDK (an immature form of the enzyme containing P-clusters but lacking FeMo-co) to obtain mature, active NifDK. Thus, within characterized diazotrophs, NifEN serves as irreplaceable scaffold for FeMo-co biosynthesis (Fig. 1) (12, 19).

Since all known diazotrophs contain Mo nitrogenases, these six *nif* genes are regarded as the minimum set for biological nitrogen fixation and can be used as an in silico tool to identify new diazotrophs (3). Interestingly, certain potential diazotrophic thermophiles deviate from this rule due to the absence of *nifE* and/or *nifN* genes, suggesting the existence of alternative, more parsimonious pathways for FeMo-co biosynthesis (20). Although there is no direct evidence showing that thermophiles lacking both *nifE* and *nifN* can fix N_2_, or that their NifDK proteins carry the canonical FeMo-co, it has been proposed that these environments may have favored primitive lineages with FeMo-co biosynthetic pathways independent of NifEN.

Ancestral NifDK lineages at the most basal position of the nitrogenase phylogenetic tree have been classified as uncharacterized nitrogenases (4, 21–23). Although these proteins contain conserved motifs typical of Mo-dependent nitrogenases, they exhibit differences in the amino acid environments surrounding FeMo-co and the P-cluster that may confer distinct maturation or catalytic properties (22, 23). At least three species of the *Chloroflexi* phylum carry *nifDK* genes of the uncharacterized nitrogenase lineage. Since species in this lineage lack NifEN, it is possible that their putative nitrogenases evolved prior to the proposed duplication and divergence of the ancestor genes that led to the *nifEN* and *nifDK* genes (24–26). The hypothesis is that, for such nitrogenases, FeMo-co biosynthesis would start with NifB activity, and its NifB-co product would be matured to FeMo-co directly in an apo-NifDK variant (an immature form containing P-clusters but lacking FeMo-co) with both scaffolding and catalytic capabilities (Fig. 1). *Roseiflexus* sp. RS-1 is one of these potential diazotrophs, carrying a basic *nifHBDK* gene cluster predicted to encode a Mo nitrogenase but lacking *nifEN*. Interestingly, diazotrophic growth of *Roseiflexus* species has not been demonstrated (27, 28), raising the question of whether or not Mo nitrogenase assembly is possible in the absence of NifEN.

In this study, we show that the predicted nitrogenase from *Roseiflexus* sp. RS-1 (hereafter *Roseiflexus*) is indeed a Mo-dependent nitrogenase in which FeMo-co is synthesized by a simpler pathway that does not require the NifEN scaffold. To this end, the *Roseiflexus* NifH, NifB, and NifDK proteins were expressed and purified from *Escherichia coli* cells, their [FeS] clusters and activities were characterized, and a complete FeMo-co biosynthetic pathway was reconstituted in vitro using only the purified proteins and their necessary substrates.

## Results

### NifDK*^RS^* Amino Acid Sequence and Structure Correspond to a Mo Nitrogenase.

A comparative amino acid sequence analysis between NifDK*^RS^* and NifDK*^Av^* (*A. vinelandii* and *Roseiflexus* proteins are designated with *Av* and *RS* superscripts, respectively) indicates the conservation of the putative FeMo-co environment within NifD, including the C^275^ and H^442^ ligands to FeMo-co and S^278^, which is hydrogen-bonded to the S atom of C^275^ (*SI Appendix*, Table S1 and Fig. S1, using *A. vinelandii* amino acid numbering). These residues are strictly conserved in all known nitrogenases (22, 23). Other highly conserved residues found in NifD*^RS^* include G^356^ and G^357^, required to avoid steric interactions with FeMo-co; R^96^ and R^359^, known to stabilize FeMo-co by hydrogen bonding its S atoms; H^195^ involved in proton transfer reactions; G^69^ and V^70^ that control substrate access to FeMo-co; and Q^191^and E^427^ near homocitrate. In addition, all NifD and NifK residues serving as P-cluster ligands were conserved, as well as α-G^87^ and α-G^185^, which are important to avoid steric interactions with the P-cluster (*SI Appendix*, Table S1) (29). Furthermore, NifD*^RS^* conserves residues that are unique to Mo nitrogenases (*SI Appendix*, Fig. S1).

A structural model for NifDK*^RS^* was built with ProMod3 using the empirically solved NifDK*^Av^* structure (10) as template. The model included amino acid residues 3 to 459 for NifD*^RS^* (95% coverage) and 6 to 447 for NifK*^RS^* (93% coverage). The predicted structure of NifDK*^RS^* and the known structure of NifDK*^Av^* were very similar, overlapping 1,770 residues with an average RMSD of 0.368 Å. The NifD overlay showed no significant differences except for the α-helix formed by residues 4 to 19 in NifD*^Av^*, which is absent in NifD*^RS^*, and the loops formed by residues 101 to 108 and residues 208 to 214 in NifD*^Av^* (*SI Appendix*, Fig. S2*A*). The NifK subunits were also superimposable except for three NifK*^Av^* regions that are absent in the shorter NifK*^RS^* (*SI Appendix*, Fig. S2*B*). The model showed nearly identical 4 Å environments around FeMo-co (*SI Appendix*, Fig. S2*C*) and the P-cluster (*SI Appendix*, Fig. S2*D*). Thus, both the presence of amino acid residues characteristic of Mo nitrogenases and their 3D position in the modeled structure were consistent with NifDK*^RS^* being a FeMo-co containing nitrogenase.

### Apo-NifDK*^RS^* Shows EPR Signals Characteristic of Immature P-clusters but Can Be Activated by the Simple Addition of FeMo-co.

To investigate the biochemical properties of NifDK*^RS^*, the *streptagII*-*nifD^RS^* and *nifK^RS^* genes were overexpressed together with *nifH^RS^* (to support the formation of P-clusters), and either the **Escherichia coli* isc* gene cluster or the *A. vinelandii nifUS^Av^* genes to boost [FeS] cluster biosynthesis.

NifDK*^RS^* was purified by StrepTactin affinity chromatography under strict anaerobic conditions. Sodium dodecyl sulfate polyacrylamide gel electrophoresis (SDS-PAGE) analysis of the purified protein revealed NifD and NifK bands with equal band intensities (*SI Appendix*, Fig. S3*A*). Size-exclusion chromatography estimated the molecular mass to be 220 kDa (*SI Appendix*, Fig. S3*B*), consistent with a theoretical α_2_β_2_ NifDK*^RS^* heterotetramer of 212 kDa, similar to all Mo nitrogenases described to date. NifDK*^RS^* preparations coexpressed with *nifUS^Av^* presented better protein solubility and higher purification yields (0.59 ± 0.24 mg NifDK*^RS^* · g^−1^ cell) than those coexpressed with the *isc^Ec^* gene cluster (0.22 ± 0.06 mg NifDK*^RS^* · g^−1^ cell). Pure NifDK*^RS^* preparations had brown/greenish color characteristic of [FeS] clusters. The spectra of as isolated apo-NifDK*^RS^* showed a broad peak around 400 nm split into two peaks at around 320 and 420 nm when samples were exposed to air (*SI Appendix*, Fig. S4). This is consistent with the breakdown of [Fe_4_S_4_] clusters (or more complex clusters) into [Fe_2_S_2_] clusters, indicating their sensitivity to O_2_, as described for mature nitrogenase proteins (30, 31). As *nifDK^RS^* was coexpressed with *nifUS^Av^* (or *isc^Ec^* genes) and *nifH^RS^*, a P-cluster containing but FeMo-co-deficient form of apo-NifDK*^RS^* containing 16 Fe atoms per tetramer would be expected if two pairs of [Fe_4_S_4_] clusters were correctly inserted and matured into two P-clusters. Indeed, purified apo-NifDK*^RS^* contained ca. 16 Fe atoms per tetramer (Table 1). Small differences were observed depending on the auxiliary genes used for [Fe_4_S_4_] cluster biosynthesis, with *nifUS^Av^* yielding slightly higher Fe content than the *isc^Ec^* operon. Thus, coexpression with *nifUS^Av^* rather than *isc^Ec^* was used for the remainder of the study.

**Table 1.** Iron content and molecular size of *Roseiflexus* Nif proteins

Nif protein	Fe atoms/molecule of protein	Molecular size (kDa)	Quaternary structure
apo-NifDK*^RS^*		212	Heterotetramer
with *isc^Ec^* cluster	14.2 ± 1.0		
with *nifUS^Av^*	16.6 ± 0.8		
NifH*^RS^* (with *isc^Ec^* cluster)	3.7 ± 0.9	52	Homodimer
NifB*^RS^* (with *nifUS^Av^*)	8.2 ± 2.6	32	Monomer

Fe content data are the mean ± SD of independent purifications (n = 2 for apo-NifDK*^RS^* with *isc^Ec^* and n = 4 for the rest).

**Fig. 2.**
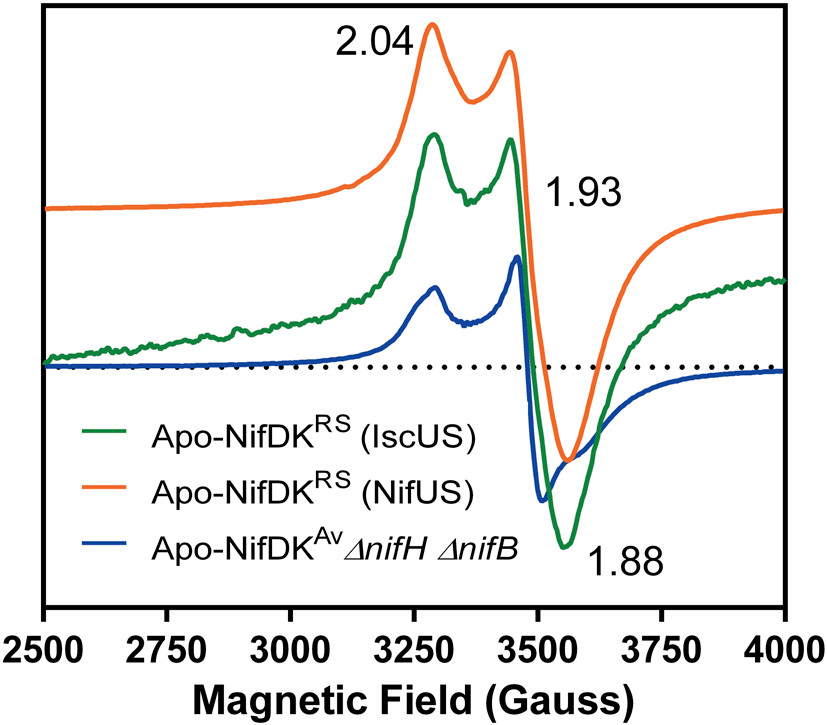
X-band EPR spectra of apo-NifDK proteins. Perpendicular mode EPR spectra of apo-NifDK*^RS^* and apo-NifDK*^Av^* in the DTH reduced states. Apo-NifDK*^Av^* was purified from a Δ*nifH* Δ*nifB A. vinelandii* strain. Pure apo-NifDK*^RS^* preparations were obtained from *E. coli* cells coexpressing NifH*^RS^* and either NifUS*^Av^* or a plasmid-cloned *isc^Ec^* gene cluster. Apo-NifDK*^RS^* (Isc*^Ec^*) spectrum was obtained from 10 scans; apo-NifDK*^RS^* (NifUS*^Av^*) and apo-NifDK*^Av^* spectra were obtained from 5 scans.

P-cluster maturation on NifDK proteins can be assessed by analyzing electron paramagnetic resonance (EPR) signals in perpendicular mode. While the immature P-clusters of a *ΔnifBΔnifH* apo-NifDK*^Av^* have an S = 1/2 EPR signal with *g* values at 2.04, 1.93, and 1.89, the dithionite (DTH)-reduced intact P-clusters are EPR silent (32–34). Apo-NifDK*^RS^* preparations coexpressed with *nifH^RS^* and *nifUS^Av^* (or *isc^Ec^* genes) exhibited EPR signals similar to those of the of *ΔnifBΔnifH* apo-NifDK*^Av^*, likely originating from immature P-clusters in the samples (Fig. 2). Similar EPR signals have been observed in samples of the Δ*nifB* apo-VnfDK. These signals were proposed to correlate either with oxidized P-clusters (35) or with heterogeneous samples containing both mature and immature P-clusters (36).

**Fig. 3.**
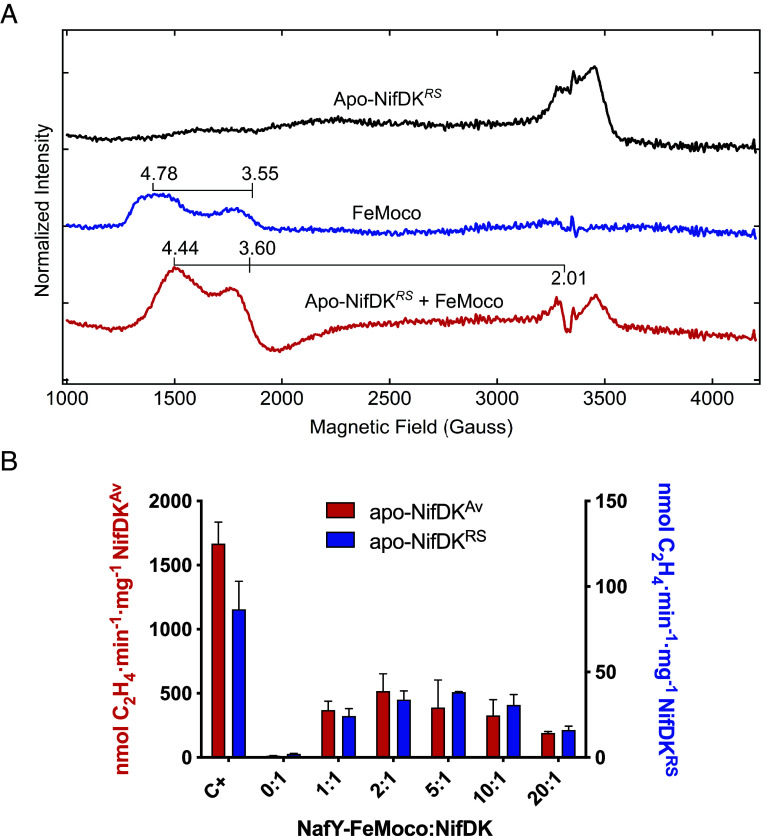
Cofactor insertion into apo-NifDK*^RS^*. (*A*) Changes in EPR signals after incubation of apo-NifDK*^RS^* with purified FeMo-co in DTH-reducing conditions. Microwave power 1 mW; temperature 5 K. Spectra were obtained from 10 scans. (*B*) Titration of apo-NifDK*^Av^* (red bars, performed at 30 °C) and apo-NifDK*^RS^* (blue bars, performed at 48 °C) reconstitution with pure NafY–FeMo-co complex (ratios indicated in the x-axis). Control reactions consisted of assays with pure NifDK*^Av^* and NifH*^Av^* at 1:40 molar ratio (C^+^ red bar), or NifB*^RS^*-dependent FeMo-co synthesis and insertion into apo-NifDK*^RS^* (composition of complete reactions in Fig. 5*A*) (C^+^ blue bar).

Apo-NifDK containing P-clusters but lacking FeMo-co (e.g., the form produced by a Δ*nifB* strain) can be reconstituted to an active form by the simple addition of purified FeMo-co in solution (9) or in complex with the *A. vinelandii* NafY carrier protein (37). Experiments of FeMo-co insertion into apo-NifDK*^RS^* were carried out by monitoring the C_2_H_2_-reducing activity of the reconstituted enzyme and the changes in EPR signals. FeMo-co in wild-type NifDK*^Av^* has a characteristic EPR signal with a distinct S = 3/2 signature at *g* = 4.3, 3.6, and 2.01. The EPR spectra of apo-NifDK*^RS^* were recorded before and after the addition of isolated FeMo-co. The emergence of a S = 3/2 FeMo-co EPR signal (*g* = 4.44 and 3.60) and the concomitant decrease in intensity of immature P-cluster EPR signal indicated the incorporation of FeMo-co into apo-NifDK*^RS^* (Fig. 3*A* and *SI Appendix*, Fig. S5).

Despite the apparent insertion of FeMo-co, the reconstituted NifDK*^RS^* protein did not show significant activity. Optimization of FeMo-co insertion into apo-NifDK*^RS^* at different temperatures was then tested. When FeMo-co insertion was carried out at 40 °C instead of 30 °C, in the presence of NifH*^RS^*, reconstituted NifDK*^RS^* produced 14.3 ± 0.3 nmol C_2_H_4_ · min^−1^ mg^−1^ NifDK*^RS^* (compared to 346 ± 42 nmol C_2_H_4_ · min^−1^·mg^−1^ produced by apo-NifDK*^Av^* reconstituted by FeMo-co at 30 °C in presence of NifH*^Av^*). To stabilize FeMo-co at higher temperatures, NafY–FeMo-co complexes were formed in vitro by incubation of NafY with FeMo-co and removal of excess FeMo-co. The highest activity of reconstituted NifDK*^RS^* (38 ± 0.4 nmol C_2_H_4_ · min^−1^ · mg^−1^ protein) was observed at 48 °C and 5-fold molar excess of the NafY–FeMo-co complex with respect to apo-NifDK*^RS^* (Fig. 3*B*), compared to the optimal 2:1 ratio of NafY to apo-NifDK*^Av^* (38). Thus, maximum activity, optimal temperature and optimal molar ratios of apo-NifDK*^RS^* to NafY–FeMo-co were different from those of the *A. vinelandii* NifDK. In conclusion, these experiments indicate sample heterogeneity, in which part of the apo-NifDK*^RS^* population could be activated by FeMo-co while another fraction contained immature P-clusters.

### Purification and Characterization of NifH*^RS^* and NifB*^RS^*.

Once it was demonstrated that apo-NifDK*^RS^* could incorporate FeMo-co, it was necessary to investigate whether NifH*^RS^*, NifB*^RS^*, and apo-NifDK*^RS^* were sufficient to assemble the active-site cofactor and reconstitute nitrogenase activity. An in vitro FeMo-co synthesis and insertion assay involving only pure *Roseiflexus* Nif proteins and the appropriate reactants was chosen to achieve unambiguous results. Thus, in addition to apo-NifDK*^RS^*, the NifH*^RS^* and NifB*^RS^* proteins were independently purified and validated prior to developing this assay. Expression, purification, and characterization of NifH*^RS^* and NifB*^RS^* are described in *SI Appendix*, Figs. S6–S17, and Tables S2–S4. It was concluded that i) NifH*^RS^* is a [Fe_4_S_4_] cluster containing homodimer of 52 kDa (*SI Appendix*, Figs. S8 and S9) with the ability to participate in P-cluster formation, FeMo-co biosynthesis, and nitrogenase catalysis, both in vitro (*SI Appendix*, Fig. S10 and Table S3) and in vivo (*SI Appendix*, Fig. S11), similar to other characterized NifH proteins (39), although in this case NifH*^RS^*did not require NifM for maturation; and ii) NifB*^RS^* is a 32-kDa monomer containing three [Fe_4_S_4_] clusters (*SI Appendix*, Figs. S14 and S15) that fulfills the known role of NifB proteins and its product, the [Fe_8_S_9_C] cluster NifB-co, in the biosynthesis of FeMo-co, both in vitro (*SI Appendix*, Fig. S16) and in vivo and (*SI Appendix*, Fig. S17) (15, 40).

### In Vitro Cofactor Synthesis for the *Roseiflexus* Nitrogenase Requires Only the Proteins NifB*^RS^*, NifH*^RS^*, and apo-NifDK*^RS^*.

**Fig. 4.**
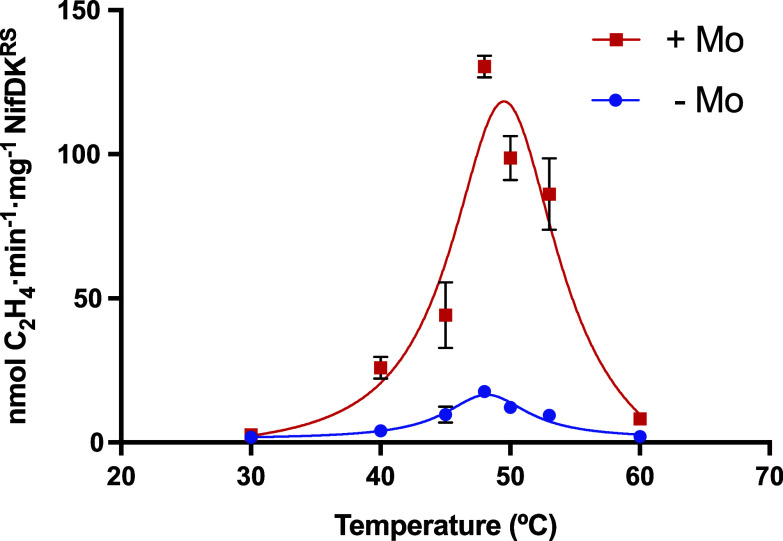
Temperature dependence of cofactor synthesis and apo-NifDK*^RS^* reconstitution. Temperature screening was performed in the presence (red) or absence (blue) of molybdate. Reaction mixtures contained NifH*^RS^*, NifB*^RS^*, apo-NifDK*^RS^*, NifX*^Av^*, homocitrate, SAM, Fe, S, Mo (when indicated), and an ATP regenerating mixture (*Materials and Methods*). Negative control assays lacking NifB*^RS^* showed negligible activity. Data represent average activity ± SD (n = 2).

Conditions for NifB*^RS^*-dependent nitrogenase cofactor synthesis and NifDK*^RS^* reconstitution were established after temperature, reaction time, and component screening assays. The complete reaction mixtures contained NifH*^RS^*, NifB*^RS^*, apo-NifDK*^RS^*, and the necessary small molecules, homocitrate, SAM, ATP, and sources of Fe, S, and Mo, under DTH reducing conditions. The NifX*^Av^* protein was added to stabilize the NifB-co formed in vitro in the initial temperature and reaction-time screenings, but it was shown not to be essential in protein component screenings (see below). All reaction mixtures lacked NifEN, an essential component for FeMo-co synthesis in all known Mo nitrogenases. Temperature screenings were performed from 30 °C to 60 °C in the presence or absence of Mo. The activity of reconstituted NifDK*^RS^* was negligible at 30 °C, reached the maximum at 48 °C (130 ± 4 nmol C_2_H_4_ min^−1^ mg^−1^ NifDK*^RS^*), and was almost inactivated at 60 °C (8.1 ± 0.4 nmol C_2_H_4_ min^−1^ mg^−1^ NifDK*^RS^*) (Fig. 4). Optimal reaction time and NifH*^RS^* to NifDK*^RS^* ratio were found at 90 min and a molar ratio of 40, respectively (*SI Appendix*, Figs. S18 and S19). It is worth noting that, at any tested temperature, much higher activities were detected in the presence of Mo than in its absence. This acetylene-reducing activity could be the result of missincorporating NifB-co or FeFe-co like cofactors as previously shown for the *A. vinelandii* apo-NifDK (41), although the presence of Mo traces in the reaction assays cannot be completely excluded. The dependency and specificity of NifDK*^RS^* on Mo was also investigated using tungsten (W) as a competitor of Mo in cofactor synthesis. W has been shown to poison Mo nitrogenase activity, probably by impairing FeMo-co biosynthesis (42). In our competition assays, cofactor synthesis was carried out in the presence of 17.5 μM molybdate and increasing concentrations of tungstate up to 1.75 mM, a 100-fold excess compared to Mo. Inhibitory effects were observed in assays with at least 50-fold excess W, in which the sequence of metal addition was unimportant (*SI Appendix*, Table S5). NifDK*^RS^* and NifDK*^Av^* showed similar sensitivity to W. These results indicate that NifDK*^RS^* is in fact a Mo nitrogenase, showing strong preference for Mo and sensitivity to W.

**Fig. 5.**
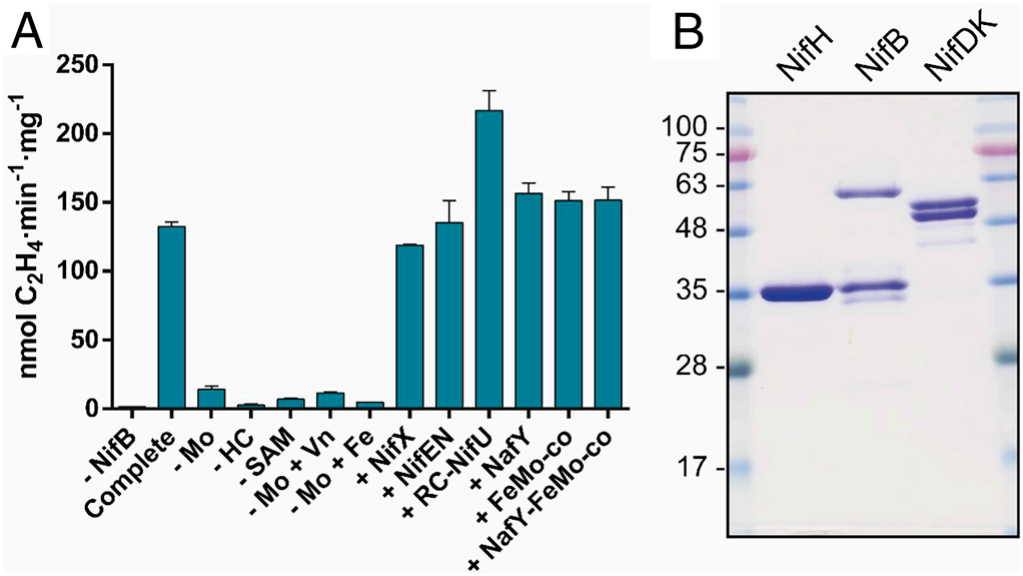
Screening of minimal components required for cofactor synthesis and apo-NifDK*^RS^* reconstitution. (*A*) Complete reactions contained molybdate, SAM, homocitrate, NifH*^RS^*, NifB*^RS^*, and apo-NifDK*^RS^*. NifX*^Av^*, NafY*^Av^*, NifEN*^Av^* or RC-NifU*^Av^* were added when indicated. RC-NifU*^Av^* refers to NifU*^Av^* loaded with [Fe_4_S_4_] clusters formed in vitro by incubation with Fe, cysteine, DTT, and NifS (*SI Appendix*, *Materials and Methods*). Reactions were performed at 48 °C. Negative control assays (- NifB) reached 1.60 ± 0.04 nmol C_2_H_4_ min^−1^ mg^−1^ NifDK*^RS^*. Data are average activities ± SD (n = 2). (*B*) SDS-PAGE analysis of purified *Roseiflexus* Nif proteins required for the in vitro synthesis of nitrogenase cofactors. NifB*^RS^* preparations contained two additional bands that were identified by mass spectrometry as GroEL (60 kDa) and C-terminal truncated NifB*^RS^* species (30 kDa).

Protein and substrate requirements for cofactor synthesis and reconstitution of NifDK*^RS^* were determined by removing or adding individual components from a complete reaction mixture containing the NifH*^RS^*, NifB*^RS^*, apo-NifDK*^RS^* proteins, and SAM, Mo, and homocitrate as substrates (Fig. 5). External sources of Fe or S were not included because previous assays indicated that NifB*^RS^* [Fe_4_S_4_] clusters fulfilled the reaction requirements (*SI Appendix*, Fig. S20). In fact, addition to the reaction mixtures of reconstituted NifU*^Av^* (RC-NifU*^Av^*) loaded with [Fe_4_S_4_] clusters boosted ethylene production (217 ± 14 nmol C_2_H_4_ min^−1^ mg^−1^ NifDK*^RS^*). It had been previously shown that NifU was a biological source of [Fe_4_S_4_] clusters for NifB to synthesize NifB-co (43, 44). Homocitrate and SAM were essential to reconstitute NifDK*^RS^*. Homocitrate is present in the active-site cofactors of all nitrogenases (10, 45–48), and SAM is required for the synthesis of NifB-co by NifB*^RS^* (15, 16, 38, 49). Consistently, adding *S*-adenosylhomocysteine (SAH) instead of SAM inhibited NifB-co formation and did not reconstitute NifDK*^RS^* (*SI Appendix*, Fig. S20). Molybdate removal resulted in lower but measurable acetylene reduction activity (14 ± 2 nmol C_2_H_4_ min^−1^ mg^−1^ NifDK*^RS^*)(Fig. 5 and *SI Appendix*, Table S5). Replacement of molybdate by vanadate or ferrous iron in the reaction mixtures did not increase the acetylene reduction activity of reconstituted NifDK*^RS^*, pointing to Mo exclusivity as designated heterometal. Addition of NafY:FeMo-co complex, or FeMo-co alone, had not effect on NifDK*^RS^* reconstitution. Addition of NifX*^Av^* or NafY*^Av^* proteins had no effect on NifDK*^RS^* reconstitution. Since these proteins bind and stabilize NifB-co and FeMo-co, respectively, their dispensability suggests efficient transfer of intermediates between NifB*^RS^* and apo-NifDK*^RS^*. Importantly, the reconstitution of NifDK*^RS^* was completely independent of NifEN (Fig. 5).

### NifDK*^RS^* Reduces N_2_ to NH_3_.

The reduction of C_2_H_2_, H^+^, and N_2_ by reconstituted NifDK*^RS^* was investigated and compared to that of the well-studied *A. vinelandii* NifDK. Reactions using NifDK*^Av^* were carried out under standard conditions using purified NifDK*^Av^* and NifH*^Av^* (30 °C and 1:40 component ratio). On the other hand, apo-NifDK*^RS^* required NifB*^RS^*-dependent cofactor synthesis to become active in substrate reduction. The conditions for NifDK*^RS^* reconstitution were established by the experiments described above and are detailed in *Materials and Methods*. Reconstitution mixtures contained NifB*^RS^*, RC-NifU*^Av^*, NifH*^RS^*, and apo-NifDK*^RS^* together with SAM, molybdate and homocitrate, and were incubated for 90 min at 48 °C. The substrate reducing activities of reconstituted NifDK*^RS^* were determined at 48 °C and with 40-fold molar excess of NifH*^RS^*. The data in Table 2 show that NifDK*^RS^* is indeed a nitrogenase as it is capable of reducing N_2_ to NH_3_. The inhibition of H^+^ reduction by N_2_ is consistent with the simultaneous reduction of N_2_ and H^+^, as occurs in the well-characterized *A. vinelandii* Mo nitrogenase (50). Although the activities for the reduction of different substrates by NifDK*^RS^* were lower than those of NifDK*^Av^*, the consistent ratios for NH_3_, C_2_H_4_, and H_2_ formation, and the almost identical selectivity for N_2_ reduction, as reflected by the NH_3_:H_2_ ratio under 1 atm N_2_, strongly suggest the presence of FeMo-co in NifDK*^RS^* and therefore its formation in the in vitro system independent of NifEN.

**Table 2.** Ethylene, hydrogen, and ammonia production activities of *A. vinelandii* and *Roseiflexus* Mo nitrogenases

	Substrate				
	N_2_			Ar	C_2_H_2_
Product	NH_3_	H_2_	NH_3_/H_2_	H_2_	C_2_H_4_
NifDK*^Av^*	849 ± 58	716 ± 103	1.19	2,123 ± 233	1,835 ± 38
NifDK*^RS^*	149 ± 30	122 ± 32	1.22	443 ± 50	391 ± 55

Activities are nmol produced per min and mg of the corresponding NifDK. H_2_ production was determined in Ar or N_2_ atmospheres. Data are the average of n = 4 independent reactions (± SD). Values from negative control reactions lacking NifDK components were subtracted. *A. vinelandii* reactions were carried out at 30 °C by mixing NifDK*^Av^* and NifH*^Av^* in molar ratio of 1:40. *Roseiflexus* reactions were carried out at 48 °C by mixing apo-NifDK*^RS^* and NifH*^RS^* in molar ratio of 1:40, together with NifB*^RS^*, RC-NifU*^Av^*, molybdate, homocitrate, and SAM.

## Discussion

We have characterized a unique Mo nitrogenase that functions both as a maturase for the synthesis of its own active-site cofactor and as a functional nitrogenase. In vitro biochemical assays demonstrated that NifH*^RS^*, NifB*^RS^*, and apo-NifDK*^RS^*, along with the substrates homocitrate, molybdate, and SAM are sufficient to synthesize a NifDK*^RS^* that effectively reduces N_2_, C_2_H_2_, and H^+^. The ratios of substrate reduction by the *Roseiflexus* nitrogenase are characteristic of a Mo nitrogenase. Significant NH_3_ production, which accounted for one-third of C_2_H_4_ production, was determined. Additionally, nitrogenase substrate preference was assessed by observing differences in H_2_ production in the presence of inert Ar or in competing N_2_. The *Roseiflexus* Mo nitrogenase exhibits a clear thermophilic tendency, as indicated by the requirement for high temperatures in cofactor synthesis and activity assays, which aligns with the bacterium’s lifestyle (28).

This study provides evidence that the *Roseiflexus* nitrogenase utilizes FeMo-co. This is supported by several observations: i) Its synthesis is dependent on Mo and homocitrate, and it is inhibited by W but not activated by V. ii) The synthesis is initiated by the SAM-radical reaction of NifB*^RS^*. iii) The product of NifB*^RS^* must be the [Fe_8_S_9_C] cluster NifB-co because it restores the Nif^+^ phenotype of a *K. oxytoca* Δ*nifB* mutant, which only contains Mo nitrogenase genes. iv) Addition of pure FeMo-co to the P-cluster containing apo-NifDK*^RS^* reconstitutes its acetylene reduction activity. v) NifDK*^RS^* has amino acid residues that are only conserved in Mo nitrogenases. vi) The ratios for NH_3_, C_2_H_4_, and H_2_ formation, and the selectivity for N_2_ reduction, are consistent with Mo nitrogenases and not with hybrid NifDK nitrogenases containing alternative FeV-co or FeFe-co.

This study presents the first example of complete in vitro FeMo-co synthesis without the NifEN maturase. This introduces a new paradigm in nitrogenase biosynthesis and challenges the well-established principle of minimal gene requirement (*nifHDKBEN*) for a functional Mo nitrogenase (3, 38). The remarkable independence of NifEN confirms the existence of an alternative and simplified pathway for FeMo-co synthesis, which has been previously hypothesized for the uncharacterized nitrogenase clade (21, 22, 24, 26). This lineage of nitrogenase is found in the earliest branches of nitrogenase evolution and is currently present in *Chloroflexi* of the *Roseiflexus* genus. These organisms possess only the *nifHBDK* genes (22), but there is no evidence of their ability to grow diazotrophically (27, 28). It is important to mention that a recently identified class of nitrogenase-like homologs encoded by closely related deep-branching nitrogen fixation-like genes were linked to ethylene and methane reactions (51).

The inherent maturase and nitrogenase activities of NifDK*^RS^* provide insight into the characteristics of ancestral nitrogenases prior to the proposed duplication and divergence of the *nifEN* and *nifDK* ancestral genes, which occurred approximately 2.1 billion years ago (26). It appears that an ancient Mo nitrogenase, which existed approximately 3.2 billion years ago (52), may have performed N_2_ fixation prior to the evolution of the canonical FeMo-co biosynthetic pathway that includes NifEN. This is currently represented by the nitrogenase from *Roseiflexus* sp. RS-1.

An ongoing debate in nitrogenase evolution is whether maturases (NifEN) are evolutionarily derived from nitrogenases (NifDK) (26, 53, 54) or, conversely, whether extant nitrogenases are derived from a primitive maturase clade (25). A recent study based on sites that are enriched for positions contributing to the functional divergence between nitrogenases and maturases suggests that canonical NifDK sequences are derived from ancestral proteins more similar to extant NifEN maturases than nitrogenase homologs (25). The authors identified twenty-three sites conserved only in nitrogenases. All of them are present in the NifDK*^RS^* protein (*SI Appendix*, Table S1). A clear example is the presence of amino acid residue H^442^ (H^422^ in NifD*^RS^*) that binds to the Mo atom of FeMo-co. Other residues close to the active site cofactor show specificity for each nitrogenase class (Mo/V/Fe) (23). Many residues characteristic of Mo nitrogenases are conserved in NifD*^RS^*, consistent with our biochemical studies demonstrating the Mo dependence of NifDK*^RS^* reconstitution. For example, R^96^ is conserved in the NifD phylogeny and is also present in the uncharacterized nitrogenase group and in NifD*^RS^* as R^85^ (*SI Appendix*, Table S1). R^96^ is known to play a role in FeMo-co reactivity (55). In contrast, both AnfD and VnfD have a lysine residue at this specific position (23).

The biochemical results of this study, supported by in silico NifDK*^RS^* characterization, indicate that an ancient diazotrophic pathway with a NifDK component integrating FeMo-co maturation and nitrogenase activity is still present in *Roseiflexus* sp. RS-1, and probably other related species living in unique ecosystems where they may play relevant biological roles. Although these diazotrophs may seem insignificant when compared to the total diazotroph pool in the biosphere, they are not only important in their habitats but also relevant as a biotechnological tool to alleviate the nitrogen crisis that the planet is facing today. The *Roseiflexus* nitrogenase is encoded by an unusually simple *nifHBDK* operon, which would facilitate its genetic transfer to relevant crops, allowing them to use atmospheric N_2_.

## Materials and Methods

### Purification of His-NifH*^RS^*, Strep-Tagged-NifB*^RS^*, and Strep-Tagged-NifDK*^RS^*.

Purification procedures were conducted using an AKTA Prime FPLC apparatus (GE Healthcare) inside a glove box with <0.1 ppm of O_2_ (MBraun). The His-NifH*^RS^* purification protocol used cells resuspended in anaerobic buffer A (100 mM Tris-HCl pH 8, 100 mM NaCl, 10% glycerol, and 2 mM DTH). The resulting CFE was loaded at 2 mL/min onto a 5 mL IMAC HiTrap Co^2+^ column previously equilibrated with buffer A following the manufacturer’s instructions (GE Healthcare). The column was then washed by 5 column volumes (CV) of buffer A and 5 CV of buffer A supplemented with 10 mM imidazole (buffer B). Elution was performed using a gradient from 10 to 300 mM imidazole (buffer C) in a 100 mL volume (*SI Appendix*, Fig. S8). Pure protein fractions were selected based on SDS-PAGE analysis and were concentrated in 30-kDa centrifugal filter units (Millipore Sigma). Concentrated pure protein was desalted using PD-10 desalting columns (Cytiva Life Sciences) previously equilibrated in buffer A.

NifB*^RS^* and NifDK*^RS^* purifications were based on a common standard protocol. Cells were resuspended using anaerobic buffer A (100 mM Tris-HCl pH 8.3, 200 mM NaCl, 10% glycerol, and 2 mM DTH), although the NifB*^RS^* purification required additional supplementation with 5 mM β-mercaptoethanol. The CFE was loaded at a flow rate of 2 mL/min onto a previously equilibrated 5-mL StrepTactin-XT HiTrap column (IBA Life Sciences). The column was washed with 20 CV of washing buffer consisting of 100 mM Tris-HCl pH 8, 200 mM NaCl, 10% glycerol, and 2 mM DTH (buffer B). Proteins were eluted using 15 mL of buffer C consisting of 100 mM Tris-HCl pH 8, 200 mM NaCl, 10% glycerol, 2 mM DTH, and 50 mM D-Biotin. Pure NifB*^RS^* (*SI Appendix*, Fig. S3) and NifDK*^RS^* (*SI Appendix*, Fig. S14) were concentrated using 30-kDa and 100-kDa centrifugal filter units, respectively. Concentrated pure proteins were desalted using PD-10 desalting columns previously equilibrated in buffer B.

### NifDK*^RS^* EPR Spectroscopy.

EPR analysis of NifDK*^RS^* was performed by continuous-wave X-band EPR spectra using a Bruker ESP-300 spectrometer with an EMX PremiumX microwave bridge and an EMXPLUS standard resonator in perpendicular mode. The system was equipped with an Oxford Instruments ESR900 continuous helium flow cryostat using VC40 flow controller for helium gas. Spectra were recorded in the 12 to 5 K temperature range, with a microwave frequency of around 9.38 GHz, microwave power of 20 mW (at 12 K) or 1 mW (at 5 K), modulation frequency of 100 kHz, modulation amplitude of 8.14 G, and time constant of 20.48 ms. If not specified, each spectrum is the sum of 10 scans and is presented after subtracting the cavity background signal recorded with an EPR tube with frozen 150 mM MOPS buffer at pH 7.3.

### In Vitro FeMo-co Synthesis and NifDK*^RS^* Reconstitution.

Assays were performed in 100 μL reaction mixtures containing 20 μM NifB*^RS^*, 3.0 μM NifH*^RS^*, 0.6 μM apo-NifDK*^RS^*, 17.5 μM Na_2_MoO_4_, 175 μM *R*-homocitrate, 125 μM SAM, and ATP regenerating mixture (22 mM Tris-HCl buffer, pH 7.5, supplemented to 1.23 mM ATP, 18 mM phosphocreatine, 2.2 mM MgCl_2_, 3 mM DTH, 40 μg/mL creatine phosphokinase, and 1 mg/mL bovine serum albumin). Optimal assay conditions were established at 48 °C for 90 min. When indicated, the reaction mixtures were modified to include, remove, or replace some components, for example, 3.0 μM NifX*^Av^*, 1.5 μM apo-NifEN*^Av^* (a form of NifEN containing two structural [Fe_4_S_4_] clusters but lacking bound FeMo-co precursors), 1.2 μM NafY*^Av^*, 1.2 μM FeMo-co, 80 μM RC-NifU*^Av^*, 500 μM SAH, 125 μM FeSO_4_, 125 μM Na_2_S, 1 mM NaVO_3_, and Na_2_WO_4_ up to 1.75 mM.

To determine the acetylene reduction activity of reconstituted NifDK*^RS^*, reaction mixtures were transferred to 9-mL sealed vials containing 24 μM of NifH*^RS^* (40-fold molar excess to apo-NifDK*^RS^*) in 500 μL of ATP regenerating mixture in 94% Ar / 6% C_2_H_2_ atmosphere. After 15 min incubation at 48 °C, reactions were stopped with 100 μL 8 M NaOH.

To measure H^+^ reduction activity of reconstituted NifDK*^RS^*, reaction mixtures were transferred to 9-mL sealed vials containing 24 μM of NifH*^RS^* in 500 μL of ATP regenerating mixture under 100% N_2_ or 100% Ar atmosphere. After 30 min incubation at 48 °C, reactions were stopped with 100 μL of 0.5 M ethylenediaminetetraacetic acid (EDTA).

To determine the N_2_ reduction activity of reconstituted NifDK*^RS^*, assays were carried out in 100 μL reaction mixes containing 10 μM NifB*^RS^*, 40 μM RC-NifU*^Av^*, 3.0 μM NifH*^RS^*, and 0.6 μM apo-NifDK*^RS^*, 125 μM SAM, 17.5 μM Na_2_MoO_4_, 175 μM *R*-homocitrate, and ATP-regenerating mixture in 100 mM MOPS pH 7.5. Samples were incubated for 90 min at 48 °C, and then transferred to 9-mL sealed vials containing 500 μL of ATP regenerating mixture supplemented with 24 μM of NifH*^RS^* in 100% N_2_ atmosphere. After 1 h incubation at 48 °C, reactions were stopped with 100 μL 0.5 M EDTA.

### FeMo-co Insertion into apo-NifDK*^RS^*.

FeMo-co was isolated from pure NifDK*^Av^* using NMF as solvent (9), and the NafY–FeMo-co complex was generated as described in ref. 37. FeMo-co insertion assays were performed in 200 μL reaction mixtures by adding either 10 μM FeMo-co in NMF or different amounts of the NafY-FeMo-co complex (0 to 12 μM) to 0.6 μM apo-NifDK*^RS^* in 100 mM MOPS pH 7.5. Reactions were incubated for 20 min at 48 °C and then transferred to 9-mL sealed vials for acetylene reduction determinations as described above.

### C_2_H_4_, NH_3_, and H_2_ Quantification.

The formation of C_2_H_4_ was quantified by gas chromatography in a Shimadzu GC-2014 equipped with a flame ionization detector (Shimadzu Corporation) coupled to a Porapak N 80/100 column (Agilent Technologies). H_2_ was quantified by gas chromatography in a Shimadzu GC-2014 equipped with a TCD detector and a 2-meter-long molecular sieve 5A 60/80 column (Agilent Technologies). Detector and column temperatures were 150 °C and 50 °C, respectively. Depending on the experiment, 25 mL/min N_2_ or Ar was used as carrier gas. Ammonia was quantified by fluorometry. Samples were filtered in 10-kDa centrifugal filter units, and 50-μL flow-through aliquots were mixed with 200 μL of Fluoraldehyde o-Phthalaldehyde reagent Solution (Thermo Scientific) and added to 96-well fluorescence detection plates. Samples were excited at 390 nm and emission was recorded at 472 nm using a VarioSkan Lux 96-well reader (Thermo Scientific). Background signal (initial fluorescence value at t = 0) was subtracted from each reaction as described (56).

## Supplementary Material

Appendix 01 (PDF)

## Data Availability

All study data are included in the article and/or *SI Appendix*.
