# Supplementary material for: Iron-molybdenum cofactor synthesis by a thermophilic nitrogenase devoid of the scaffold NifEN
Source: Proc Natl Acad Sci U S A. 2024 Nov 6;121(46):e2406198121. doi: 10.1073/pnas.2406198121 (PMC11573651; doi:10.1073/pnas.2406198121)
Supplement: Supplementary file 1 — Appendix 01 (PDF) [file pnas.2406198121.sapp.pdf]

SUPPORTING INFORMATION

Iron-molybdenum cofactor synthesis by a thermophilic nitrogenase devoid of the scaffold NifEN

Lucía Payá-Tormo[1,2], Carlos Echavarri-Erasun,[1,2*] Natalia Makarovsky-Saavedra,[1,2] Ana Pérez-González,[1] Zhi-Yong Yang,[3] Yisong Guo,[4] Lance C. Seefeldt,[3] and Luis M. Rubio[1,2*]

[1] Centro de Biotecnología y Genómica de Plantas (UPM-INIA/CSIC), Campus Montegancedo, 28223 Pozuelo de Alarcón (Madrid), Spain.

[2] Departamento de Biotecnología-Biología Vegetal, Escuela Técnica Superior de Ingeniería Agronómica, Alimentaria y de Biosistemas, Universidad Politécnica de Madrid, 28040 Madrid, Spain.

[3] Department of Chemistry and Biochemistry, Utah State University, Logan, Utah, USA

[4] Department of Chemistry, Carnegie Mellon University, Pittsburgh, PA 15213, USA

*Correspondence: Luis M. Rubio (luis.rubio@csic.es); Carlos Echavarri-Erasun (carlos.echavarri@upm.es).

SUPPORTING RESULTS

NifH^{RS} conserves key amino acid residues for [Fe_4S_4] binding and NifDK interaction. Amino acid sequence analysis of NifH^{RS} (UniProt accession number A5USK5) revealed conservation of consensus residues present in characterized NifH proteins (Figure S6 and Table S2). In particular, NifH^{RS} conserves the cysteine residues that bind the [Fe_4S_4] cluster and other residues that play an important role in stabilizing the complex with NifDK during electron transfer (*e.g.* R^{98}, which corresponds to the R^{100} in NifH^{Av}) (1, 2). Other residues involved in NifDK protein interaction (*e.g.* E^{112}) were not conserved, which is not unusual as some residues important for complex formation are species-specific (1). A structural model of NifH^{RS} constructed using as template NifH from *Methanosarcina acetivorans* (PDB ID 6NZJ)(3), showed that the two structures were virtually indistinguishable (all residues overlapping with an RMSD of 0.181 Å), except for the A^{96} and G^{96} in NifH^{Ma} and NifH^{RS}, respectively, which are located in a 4 Å environment around the [Fe_4S_4] cluster (Figure S7).

NifH^{RS} is a homodimer containing one [Fe_4S_4] cluster. His-tagged-NifH^{RS} (called NifH^{RS} for simplicity) was purified from heterologous *E. coli* BL21 (DE3) cells overexpressing the *isc* genes for enhanced [FeS] cluster biosynthesis (4). Co-expression with *nifM* was not necessary because NifH^{RS} has been shown to be NifM-independent (5). NifH^{RS} was purified to homogeneity under strict anaerobic conditions in buffers containing sodium dithionite (DTH) as reducing agent (Figure S8A). The identity of purified NifH^{RS} was confirmed as RoseRS_1201 by mass spectrometry. Size exclusion chromatography yielded native molecular mass of 52 kDa protein, consistent with a homodimer structure (Figure S8B). The Fe content was 3.7 ± 0.9 atoms per NifH^{RS} dimer (Table 1). The UV-visible spectrum of *as isolated* NifH^{RS} presented a shoulder at 315 nm and a broad peak around 400 nm indicative of [Fe_4S_4] clusters (6). Exposure to air resulted in the disappearance of the 400 nm peak and the appearance of a 400-430 nm peak consistent with degradation of [Fe_4S_4] into [Fe_2S_2] clusters (Figure S9A), indicating sensitivity to O_2 as described for other NifH proteins (7, 8). The EPR spectrum of DTH-reduced NifH^{RS} presented a S = 1/2 rhombic signal in the $g \approx 2$ region with g-values of 2.01, 1.94, 1.85, and a S =3/2 EPR signal around $g \approx 5$ (Figure S9B). It resembled EPR spectra of *A. vinelandii, K. oxytoca* and *C. pasteurianum* NifH proteins found in the paramagnetic 1^+ oxidation state ($[Fe_4S_4]^{1+}$) with 3 Fe^{2+} and 1 Fe^{3+} atoms (9-11). A signal detected around g = 4.3 could arise from adventitious iron (9).

NifH^{RS} exhibits the three activities characteristic of NifH. NifH^{RS} dinitrogenase reductase activity was confirmed by titrating NifDK^{Av} acetylene reduction activity (Figure S10). A 200-

fold molar excess of NifH^{RS} was required for maximal activity. NifH^{RS} also supported N_2 reduction to NH_3 by NifDK^{Av} (Table S3). Additionally, NifH^{RS} demonstrated activity in the *in vitro* FeMo-co synthesis assay in which purified NifB-co is converted into FeMo-co and inserted into apo-NifDK^{Av} to reconstitute nitrogenase activity in reactions that require molybdate, homocitrate, ATP, NifX^{Av}, apo-NifEN^{Av}, and apo-NifDK^{Av} (Table S3). Finally, the capacity of NifH^{RS} to assemble the P-clusters was demonstrated through the *in vitro* reconstitution of nitrogenase activity of apo-NifDK^{Av} containing immature P-clusters, for which NifH^{RS} and apo-NifDK^{Av} were incubated prior to the insertion of FeMo-co in the presence of ATP regenerating mixture (Table S3). Reactions using NifH^{Rs} in the P-cluster maturation step and NifH^{Av} in the acetylene reduction showed 5-fold more activity than reactions using NifH^{Rs} in both steps. The lower activities observed for NifH^{RS} in comparison to NifH^{Av} may be attributed to the low assay temperature (30 ºC) and suboptimal interactions of the former with the *A. vinelandii* components required for P-cluster maturation, FeMo-co synthesis, and nitrogenase activity.

NifH^{RS} supports diazotrophic growth in *A.vinelandii*. The NifH^{RS} participation in P-cluster and FeMo-co formation, and its compatibility with NifDK^{Av}, were further substantiated by the capacity to restore diazotrophic growth of an *A. vinelandii nifH* deletion mutant (Figure S11). The *A. vinelandii* DC127 strain carries a *nifH^{RS}* gene in place of *nifH^{Av}*. It additionally lacks *vnfH* to prevent compensation of NifH function by VnfH (12). NifH^{RS} supports *A. vinelandii* diazotrophic growth, albeit not as strongly as the native NifH^{Av}, in alignment with the results of the *in vitro* assays.

NifB^{RS} conserves key amino acid residues important for [Fe_4S_4] cluster binding and activity. Amino acid sequence analysis of NifB^{RS} (UniProt accession number A5USK4) revealed conservation of consensus residues present in characterized NifB proteins, including the *Methannotrix thermoacetophila* NifB^{Mt} (UniProt accession number A0B690), which 3D structure has been determined by protein crystallography (13). NifB^{RS} conserves the radical SAM specific motif that coordinates the RS [Fe_4S_4]-SAM cluster, and motifs to coordinate two additional [Fe_4S_4] clusters, called K1 and K2, that serve as substrates and are fused to generate the [Fe_8S_9C] NifB-co (Figure S12 and Table S4). A structural model of NifB^{RS} constructed using as template NifB^{Mt} (PDB ID 6Y1X) (13), showed that the two structures were virtually indistinguishable (87% sequence coverage and overlap with an RMSD of 0.111 Å), except for slight differences 4 Å around the RS and K1 clusters (Figure S13).

NifB^{RS} is a monomer containing three [Fe_4S_4] clusters. Purified preparations of twin-strep-

tagged NifB^{RS} (called NifB^{RS} for simplicity) were obtained from recombinant *E. coli* BL21 (DE3) cells co-expressing the *A. vinelandii nifUS^{Av}* genes by anaerobic Strep-Tactin affinity chromatography. Purified NifB^{RS} appeared as a 30-35 kDa doublet on SDS-PAGE suggesting some level of protein degradation (Figure S14A). Mass spectrometry confirmed that both bands were NifB^{RS} (RoseRS_1200), the lower band being a C-terminal truncated species (Figure S14B). The band observed at 60 kDa was identified as GroEL, indicating poor solubility of overexpressed NifB^{RS} in *E. coli*. Size exclusion chromatography revealed native molecular mass of 32 kDa consistent with a monomeric structure (Figure S14C). As isolated NifB^{RS} monomers contained an average of 8.2 ± 2.6 Fe atoms (Table 1). The UV-visible spectrum of *as isolated* NifB^{RS} preparations showed a profile common to other [Fe_4S_4] cluster-containing proteins and O_2-dependent transitions indicative of cluster degradation (Figure S15A). NifB^{RS} exhibited EPR signals from its three paramagnetic $[Fe_4S_4]^{+1}$ clusters (Figure S15B), as it had been previously reported for *Methanocaldococcus infernus* and *Methanobacterium thermoautotrophicum* NifB proteins (14, 15). Therefore, spectroscopic analysis concluded that purified NifB^{RS} preparations contain all the metal clusters required for NifB activity albeit not at full occupancy.

NifB^{RS} functions in the *in vitro* FeMo-co synthesis assay. NifB^{RS}-dependent FeMo-co synthesis and insertion into apo-NifDK was determined in the assay established by Curatti using proteins purified from *A. vinelandii* (with the exception of NifB^{RS}) (16). Complete reactions containing NifB^{RS}, NifX^{Av}, apo-NifEN^{Av} (a form of NifEN containing two structural [Fe_4S_4] clusters but lacking bound FeMo-co precursors), NifH^{Av}, apo-NifDK^{Av}, ATP, homocitrate, SAM, molybdate, $FeSO_4$, and Na_2S were able to reconstitute nitrogenase activity, with NifB^{RS} being able to fulfill the role of NifB-co. NifB^{RS} obtained from recombinant *E. coli* cells co-expressing *nifU^{Av}*, *nifS^{Av}* and *fdxN^{Av}* was slightly more effective than NifB^{RS} produced in the absence of *fdxN^{Av}* (Figure S16).

NifB^{RS} restored diazotrophic growth of a *K. oxytoca nifB* deletion mutant. *K. oxytoca* UC9 (*ΔnifB*) (17) was used in a genetic complementation study to investigate the function of NifB^{RS} *in vivo*. A his-tagged version of *nifB^{RS}* was cloned under the control of the IPTG-inducible *trc* promoter and used to transform UC9. Diazotrophic growth and *in vivo* acetylene reduction activity were measured for 72 h following nitrogenase derepression and IPTG induction (Figure S17A and S17B). Wild type *K. oxytoca* (UN strain) showed maximum nitrogenase activity after 8 h of derepression (9.2 ± 0.2 nmol $C_2H_4 \cdot min^{-1} \cdot OD^{-1}$) and grew to an OD_{600nm} of 2. The UC9 strain transformed with his-*nifB^{RS}* required 48 h of diazotrophic growth

for maximum nitrogenase activity (3.4 ± 0.6 nmol $C_2H_4 \cdot min^{-1} \cdot OD^{-1}$) and grew to an OD_{600nm} of 1.1. Thus, nitrogenase activity in the complemented strain was 37% of that of the wild-type strain. Maximum nitrogenase activity correlated with His-NifB^{RS} protein expression (Figure S17C). UC9 transformed with the expression vector lacking *nifB*^{RS} showed neither acetylene reduction activity nor diazotrophic growth.

SUPPORTING MATERIALS AND METHODS

Bacterial strains and plasmids. *E. coli* DH5α and BL21 (DE3) strains were used for cloning and Nif protein overexpression, respectively. Plasmids constructed in this work are shown in Table S6. The *Roseiflexus sp.* RS-1 *nif* gene cluster was synthesized by GeneScript (New Jersey, USA) with codon optimization for use in *E. coli* and cloned into the *Nde*I and *BamH*I sites of pET16b to generate pRHB513.

NifH^{RS} (RoseRS_1201) was overexpressed from pRHB513 as an N-terminal His-tagged NifH^{RS} fusion. NifB^{RS} (RoseRS_1200) was overexpressed from pN2LP123, a modified pET16b vector in which the His-tag encoding sequence was replaced with a sequence encoding a double *streptagII* (TwinStreptagII) followed by a TEV-protease recognition site. Then, *nifB*^{RS} was cloned into the modified pET16b vector using *Nde*I and *BamH*I restriction sites. NifDK^{RS} was overexpressed from pN2LP49. The *nifD*^{RS} (RoseRS_1199) and *nifK*^{RS} (RoseRS_1198) genes were amplified together from pRHB513 using Phusion Hot Start II High-Fidelity DNA Polymerase (Thermo Fisher Scientific) with the primers: 5′-CAGGAGCTCATGTGGTCTCATCCGCAGTTTGAAAAAATGCAGTTCAAATGCAATCAG-3′ and 5′-CAGAAGCTTCTAACCGTGAGCCGTGG-3′. A sequence encoding a StrepTagII at the 5' end of *nifD*^{RS} was included in the forward primer to generate a StrepTagII-NifD^{RS} fusion. PCR resulted in a 2,855 bp DNA fragment, which was then inserted into the *Sac*I and *Hind*III sites of vector pTRC99a to generate pN2LP49.

In some instances, the overexpression of Nif proteins in *E. coli* require additional proteins involved in [FeS] cluster biosynthesis (15, 18). Three pRSF-*isc-metK*-Duet-1 derivative plasmids were constructed to facilitate Nif protein expression. In pN2LP30 the *E. coli isc* gene cluster located between the *Nco*I and *Not*I sites was replaced by the *A. vinelandii nifUS* sequences amplified with oligonucleotides: 5′-TTAATAAGGAGATATACCATGGCCTGGGATTATTCGGAAA-3′ and 5′-TTCGACTTAAGCATTATGCGGCCGCTCAGCCGTAGACCGG-3′ using plasmid

pRHB608 as template (13). Both primers contained 15-base pair extensions complementary to the target vector for ELIC cloning of *nifU*^{Av} and *nifS*^{Av} genes (19). Plasmids pN2LP50 and pN2LP51 were derivatives of pRSF-*isc*-*metK*-Duet-1 and pRSF-*nifUS*-*metK*-Duet-1, respectively, in which the *metK* gene was replaced by *nifH*^{RS} using the *Nde*I and *Xho*I restriction sites.

DNA digestions were performed with New England Biolabs restriction enzymes; ligations were performed with Promega T4 ligase; PCR amplifications were performed using Phusion Hot Start II High-Fidelity DNA Polymerase. *E. coli* competent cells were transformed using heat-shock and selected using appropriate antibiotics. Plasmid extractions were performed using Qiaprep Spin Miniprep kit (Qiagen), and cloning fidelity was verified by sequencing (Macrogen).

Overexpression of *Roseiflexus* proteins in *E. coli*. Transformed *E. coli* BL21 (DE3) cells containing combinations of the *nifH*^{RS}, *nifB*^{RS}, or *nifDK*^{RS} expression plasmids and supplementary vectors (pRSF-*isc*-*metK*-Duet-1, pN2LP30, pN2LP50, or pN2LP51) were grown in LB liquid media plus antibiotics for 2-3 hours at 37 ºC and 200 rpm until an OD_{600nm} of 0.6-0.8. This preculture was then used to inoculate four 4-L flasks, each containing 1 L of LB medium supplemented with 20 μM ammonium iron (III) citrate and antibiotics, to an OD_{600nm} of 0.02-0.05. Early aerobic growth conditions were established at 37 ºC and 200 rpm. When cultures reached 0.6-0.8 of OD_{600nm}, the media was supplemented with 8.7 mM lactose, 2 mM cysteine, and 0.2 mM ammonium iron (III), growth conditions were changed to 30 ºC and 105 rpm for overnight overexpression (20, 21). The next day cells were collected by centrifugation at 5,000 x *g* for 10 min at 4 ºC, and the resulting cell pellets were frozen in liquid N_2 and stored at -80 ºC.

Preparation of *E. coli* cell-free-extracts (CFE). All buffers used were made anaerobic by extensive sparging with purified N_2 followed by the addition of 2 mM DTH. Cells were resuspended in anaerobic lysis buffer composed of the corresponding buffer A, which changed depending of the protein to be purified (see Materials and Methods in main text), supplemented with 1 mM phenylmethylsulfonyl fluoride, 2.3 μM leupeptin, and 5 μg/mL DNase I, using a cell to buffer ratio (w/v) in the range of 1:1 to 1:3. Cells were lysed passing through a Emulsiflex-C5 homogenizer (Avestin) previously equilibrated with anaerobic lysis buffer using 15,000 psi disruption pressure under strict anaerobic conditions. The homogenized mixture was centrifuged at 50,000 x *g* for at least 45 min at 4 °C in dual-sealed centrifuge bottles in a Beckman JA-25.50 rotor. The resulting soluble CFE was filtered through a 0.2 μM

membrane inside a glove box to remove precipitated material before loading onto chromatography columns.

Overexpression and purification of NifH^{RS} from *Saccharomyces cerevisiae.* Strep-tagged NifH^{RS} used in the P-cluster maturation assays was obtained from *S. cerevisiae* SB321Y expressing mitochondrial targeted NifH^{RS}, NifU^{Av} and NifS^{Av} as described (5).

Purification of Strep-tagged apoNifDK^{Av} with immature P-clusters from *A. vinelandii.* DJ2106 (*ΔnifH*) cells were grown in a 240-L fermenter at 30 °C in modified Burk's medium with 5.7 mM ammonium acetate as. Once ammonium was exhausted, cells were cultured for 3 additional hours and harvested. Cells were resuspended in anaerobic 50 mM Tris-HCl pH 8.3, 500 mM NaCl, 20% glycerol, and 2 mM DTH, and purified using Strep-Tactin®XT 4Flow® high-capacity columns (IBA Life Sciences) as described (22). Purifications were conducted inside a glove box (MBraun) containing <0.1 ppm of O_2.

UV-visible spectroscopy. Samples for UV-visible spectroscopy were prepared anaerobically inside a MBraun glove box. Pure proteins were first desalted of DTH traces using buffer A (specific for each protein, see Materials and Methods in main text) using PD-10 desalting columns. Ten to 100 µL of desalted pure proteins were diluted in 700 µL of buffer A without DTH. Samples were moved into sealed quartz spectroscopy cuvettes and were scanned from 225 nm to 800 nm using a dual-beam Shimadzu UV-2600 spectrophotometer (Shimadzu, Japan). UV-visible absorption spectra were recorded using buffer A as baseline. The absorbance at 800 nm was subtracted and the spectra were normalized to 279 nm.

NifH^{RS} and NifB^{RS} EPR spectroscopy. NifH^{RS} and NifB^{RS} analysis was performed using X-band (9.64 GHz) EPR spectra recorded in a Bruker E500A spectrometer equipped with an Oxford ESR 910 cryostat for low-temperature measurements (Bruker, Massachusetts, USA). The microwave frequency was calibrated using a frequency counter, and the magnetic field was calibrated using an NMR gauss meter. The temperature of the X-band cryostat was calibrated using a carbon-glass CGR-1-1000 resistor temperature probe (LakeShore Cryotronics, Ohio, USA). For every EPR spectrum, a modulation frequency and amplitude of 100 kHz and 1 mT were used. EPR spectral simulations were performed with Spin Count (23) using a 1 mM Cu(II)ethylenediaminetetraacetic solution as spin quantification standard.

Protein methods. Protein concentration was determined using the BCA Protein Assay Kit (Pierce, USA) (24). To eliminate the interfering effect of DTH, pure protein samples were pre-treated with iodoacetamide (2 mg/mL) for 15 minutes at 37 ºC before conducting the BCA

assay. The Fe^{2+} content of purified protein samples was estimated using the bipyridyl assay (25). Absorbance at 520 nm was measured using a Shimadzu UV-2600 spectrophotometer. The procedure for SDS-PAGE has been described (26).

Size-exclusion chromatography. Purified NifH^{RS}, NifB^{RS}, and NifDK^{RS} were analyzed by size-exclusion chromatography using a HiLoad 16/600 Superdex 200 column (GE Healthcare) controlled by a AKTA Prime FPLC (GE Healthcare). The column was equilibrated with 100 mM Tris-HCl, pH 7.6, 200 mM NaCl, 10% glycerol, and 2 mM DTH at 1 mL/min. The column was calibrated for molecular mass determination using conanbunin (75 kDa), aldolase (158 kDa), *M. infernus* NifB (36 kDa), *A. vinelandii* NifH homodimer (63 kDa), *A. vinelandii* NifEN heterotetramer (202 kDa) and *A. vinelandii* NifDK heterotetramer (230 kDa).

Protein Mass Spectrometry. Protein mass spectrometry was carried out with a 4800 Proteomics Analyzer equipped with TOF/TOF mass spectrometer at the Proteomics facility of the Pharmacy School of Universidad Complutense de Madrid (Madrid, Spain).

Immunoblot analysis of NifB^{RS} expression. Proteins samples were transferred to 0.45 µm nitrocellulose membranes after SDS-PAGE using a TransBlot Semi-dry transfer device (Bio-Rad, USA). Membranes were: (*i*) blocked for 1 h at room temperature (RT) using TBS-t buffer (20 mM Tris-HCl, 150 mM NaCl, 0.02% Tween-20, pH 7.5) supplemented with 5% (w/v) non-fat dried milk; (*ii*) washed twice using TBS-t buffer for 5-10 minutes; (*iii*) incubated with anti-His antibody (1:5,000 dilution) at 4 ºC overnight in an orbital shaker. Primary antibody work solution was prepared in 5% BSA TBS-t buffer in the presence of 0.1% NaN_3. Next day, membranes were washed 3 times with TBS-t buffer for 10 minutes and incubated with anti-mouse antibody solution (1:20,000 dilution) at RT for 1 h. Secondary antibody solution was prepared in buffer TBS-t supplemented with 2% milk. Membranes were washed 3 times for 10 minutes in TBS-t buffer before developing signals by Enhance Chemiluminescence.

Reconstitution of NifU^{Av} [Fe_4S_4] clusters mediated by NifS^{Av}. Pure NifU^{Av} was isolated from *E. coli* and reconstituted *in vitro* as described (6, 27). As-isolated NifU^{Av} (20 µM) was diluted in 100 mM MOPS buffer, pH 7.5, supplemented with 8 mM of dithiothreitol (DTT) for 30 minutes at 37 ºC. The mixture was then supplemented with 1 mM L-cysteine, 225 nM of purified NifS^{Av}, and 9 mM DTT. Iron was carefully supplemented in eight portions in 15 minute intervals to reach a final $Fe(NH_4)_2(SO_4)_2\cdot 6H_2O$ concentration of 0.8 mM. The reconstitution mixture was incubated at 4 ºC for 3 hours. Iron and DTT excess were removed using 30-kDa centrifugal filter units followed by re-isolation of the reconstituted NifU^{Av} (RC-NifU^{Av}) through

a pre-packed 200 µL StrepTactin-RS column (IBA LifeScience) to remove strep-tagged NifS from the mixture.

NifH^{RS} ditrogenase reductase activity. Titration reactions contained 3 µg of pure NifDK^{Av} (13 nM NifDK^{Av}) and increasing amounts of pure NifH^{RS} (up to 300 molar excess) in 0.2 mL of 22 mM Tris-HCl pH 7.4 supplemented with 0.8 mL of ATP regenerating mixture. Control reactions were performed with NifDK^{Av} and NifH^{Av} proteins. Assays were carried out in 9-mL sealed vials under 100% Ar. Reactions were initiated by injecting 500 µL C_2H_2, proceeded for 15 minutes at 30 ºC, and were stopped with 100 µL 8 M NaOH. C_2H_4 production was measured by gas chromatography.

For NH_3 production activity, reaction mixtures contained 12 µg of NifDK^{Av} (34.8 nM) and 145.6 µg of NifH^{RS} (1.57 µM; 40-fold molar excess) in 0.2 mL of 100 mM MOPS pH 7.5 supplemented with 0.8 mL of ATP regenerating mixture, Control reactions were performed with NifDK^{Av} and NifH^{Av} proteins. Assays were carried out in 9-mL sealed vials under 100% N_2 for 30 min at 30 ºC. Reactions were stopped with 100 µL 0.5 M EDTA. Twenty-five µL of each sample were used to determine NH_3.

NifH^{RS} P-cluster maturation activity. P-cluster maturation assays were performed *in vitro* by reconstituting the nitrogenase activity of apo-NifDK^{Av} with immature P-clusters. The assays had three steps. The P-cluster maturation step was performed in 200 µL reaction mixtures containing 0.6 µM of apo-NifDK^{Av} and 3 µM NifH^{Av} or NifH^{RS} in ATP regenerating mixture. Reactions were incubated for 30 min at 30 ºC with gentle agitation (350 rpm) in a ThermoBlock (Eppendorff). After P-cluster maturation, 1.2 µM of pure FeMo-co was added to the reactions and incubated for additional 30 min at 30 ºC. Reconstitution of apo-NifDK^{Av} was stopped by addition of 10 nmol of ammonium tetrathiomolybdate ($(NH_4)_2MoS_4$, Sigma). In a last step, acetylene reduction assays for reconstituted NifDK^{Av} were performed. Reaction mixtures were transferred to 9-mL sealed vials containing NifH^{Av} or NifH^{RS} at 40-fold molar excess to apo-NifDK^{Av} and 500 µL of ATP regenerating mixture. Reactions were incubated with agitation for 15 min at 30ºC in a 94% Ar / 6% C_2H_2 atmosphere and stopped with 100 µL 8M NaOH. All steps were performed inside a glove box (CoyLabs) with <0.1 ppm of O_2.

***In vivo* genetic complementation of *A. vinelandii ΔnifH* with *nifH^{RS}*.** The *nifH^{RS}* gene was amplified from plasmid pRHB513 using primers: 5′-GAAATGCAACCTGAGGAAATTACATATGGCTATGCGTCAAGTGGCGTTCTATGGTAAAGG and 3′- ACCGGAGCGGCTATCAGACTTCTTCGTCCACAATA. The intergenic

region between *nifH*^{Av} and *nifD*^{Av} was amplified from plasmid pDB6 with primers 5′-GCGAATACGGTATTGTGGACGAAGAAGTCTGATAG and 3′-CTTGGACTGGGTAACCGCCG. Amplified fragments were used as template in Phusion PCR reactions with primers 5′-GAAATGCAACCTGAGGAAATTACATATGGCTATGCGTCAAGTGGCGTTCTATGGTAAAGG and 3′- CTTGGACTGGGTAACCGCCG. The new PCR fragment containing *nifH*^{RS} and the *nifH*^{Av} and *nifD*^{Av} intergenic region was digested with Bsu36I y BstEII and inserted into pDB6 digested with the same enzymes. The generated pASC36 plasmid has *nifH*^{RS} replacing *nifH*^{Av} in its native locus. To avoid improper *nifH*^{RS} and *nifD*^{Av} gene translation, the first and last three native codon sequences of *nifH*^{Av} were included before and after of the *nifH*^{RS} sequence. pASC36 was sequenced by Plasmidsaurus, Inc. *A. vinelandii* DC127 strain was generating by transformation of DC115 (*ΔvnfDGK*::SmR, *ΔanfHDGK*::GentR, *ΔnifHD*::KanR) with pASC36 following established protocols (28). The presence of *nifH*^{RS} replacing *nifH*^{Av} in DC124 was confirmed by DNA sequencing. Strain DC124 was subsequently transformed with pDB2080 to insert a kanamycin resistance cassette in *vnfH.*

A. vinelandii cells were plated in Burk's modified nitrogen-free medium plates (29) supplemented with 1μM sodium molybdate. For non-diazotrophic conditions, 13 mM ammonium acetate was added to the media as the nitrogen source. Strains were grown at 30ºC for 6 days.

NifH^{RS} FeMo-co synthesis activity. Assays were performed in 100 μL reaction mixes containing 2.55 μM NifB-co, 3.0 μM NifX^{Av}, 1.5 μM apo-NifEN^{Av}, 3.0 μM NifH^{RS}, and 0.6 μM apo-NifDK^{Av} supplemented with 17.5 μM Na_2MoO_4, 175 μM *R*-homocitrate and ATP regenerating mixture. Reactions for FeMo-co synthesis and insertion into apo-NifDK^{Av} were incubated at 30 °C for 45 min. Activity of reconstituted NifDK^{Av} was analyzed by the acetylene reduction assay after addition of 2.4 nmol of NifH^{RS} (1:40 ratio of NifH^{RS} to NifDK^{Av}) and 0.5 mL of ATP regenerating mixture in 9 mL sealed vials under 94% Ar / 6% C_2H_2 for 15 min at 30 ºC. C_2H_4 formation was measured by gas chromatography.

NifB^{RS}-dependent *in vitro* FeMo-co synthesis and insertion into apo-NifDK^{Av}. Assays were carried out in 100 μL reactions containing 20 μM NifB^{RS}, 125 μM $FeSO_4$, 125 μM Na_2S, 125 μM SAM, 3.0 μM NifX^{Av}, 1.5 μM apo-NifEN^{Av}, 3.0 μM NifH^{Av}, 17.5 μM Na_2MoO_4, 175 μM *R*-homocitrate, 0.6 μM apo-NifDK^{Av}, and ATP-regenerating mixture. Reactions for FeMo-co synthesis and insertion into apo-NifDK^{Av} were incubated at 30 °C for 45 min. The activity of the reconstituted NifDK^{Av} was analyzed by the acetylene reduction assay after the addition

of 1.2 nmol of NifH^{Av} (1:20 ratio of NifH^{Av} to NifDK^{Av}) and 0.4 mL of ATP regenerating mixture in 9 mL sealed vials under 94% Ar / 6% C_2H_2 for 15 min at 30 ºC. C_2H_4 formation was measured by gas chromatography.

***In vivo* genetic complementation of *K. oxytoca* UC9 (*ΔnifB*) with *nifB*^{RS}.** Plasmid pN2LP41 carries a his-tagged *nifB*^{RS} gene cloned under the control of IPTG-inducible *trc* promoter using the *Nco*I and *BamH*I sites of pN2SB73, a pTRC-99a derivative that had the ampicillin resistance cassette replaced by kanamycin resistance. The *K. oxytoca* UC9 strain (*ΔnifB nif*- phenotype) (17) was transformed with pN2LP41 for complementation or with pN2SB73 as negative control. Strains were incubated overnight at 30 ºC in minimal medium supplemented with 28.5 µM ammonium acetate (30). The following day, cells were washed three times using N-free minimal medium and were resuspended at OD_{600nm} of 0.15 in 20 mL of N-free medium supplemented with the corresponding antibiotics and 0.1% serine for nitrogenase derepression. Cultures were set up in 100 mL vials with rubber-sealed caps and air was replaced with 100% N_2. NifB^{RS} expression was induced with 25 µM IPTG. Cell growth was monitored with an Ultrospec 3300 Pro spectrophotometer (Amersham Biosciences). *In vivo* C_2H_2 reduction was determined by C_2H_4 production at 30 ºC for 30 min using 1-mL culture samples in anaerobic Ar-flushed sealed 9-mL vials. Culture samples were withdrawn for immunoblot analysis as described above.

REFERENCES

1. J. W. Peters, K. Fisher, D. R. Dean, Nitrogenase structure and function: a biochemical-genetic perspective. *Annu. Rev. Microbiol.* **49**, 335-366 (1995).
2. D. Wolle, C. Kim, D. Dean, J. B. Howard, Ionic interactions in the nitrogenase complex. Properties of Fe-protein containing substitutions for Arg-100. *J. Biol. Chem.* **267**, 3667-3673 (1992).
3. L. A. Rettberg *et al.*, Structural analysis of a nitrogenase iron protein from *Methanosarcina acetivorans*: Implications for CO2 capture by a surface-exposed [Fe_4S_4] cluster. *mBio* **10** (2019).
4. Y. Nicolet, L. Zeppieri, P. Amara, J. C. Fontecilla-Camps, Crystal structure of tryptophan lyase (NosL): evidence for radical formation at the amino group of tryptophan. *Angew. Chem. Int. Ed.* **53**, 11840-11844 (2014).
5. L. Paya-Tormo *et al.*, A colorimetric method to measure in vitro nitrogenase

functionality for engineering nitrogen fixation. *Sci. Rep.* **12**, 10367 (2022).

6. A. D. Smith *et al.*, NifS-mediated assembly of [4Fe - 4S] clusters in the N- and C-terminal domains of the NifU scaffold protein. *Biochemistry* **44**, 12955-12969 (2005).
7. R. R. Eady, B. E. Smith, K. A. Cook, J. R. Postgate, Nitrogenase of *Klebsiella pneumoniae*. Purification and properties of the component proteins. *Biochem. J.* **128**, 655-675 (1972).
8. V. K. Shah, W. J. Brill, Nitrogenase. IV. Simple method of purification to homogeneity of nitrogenase components from *Azotobacter vinelandii*. *Biochim. Biophys. Acta* **305**, 445-454 (1973).
9. W. R. Hagen, R. R. Eady, W. R. Dunham, H. Haaker, A novel S= 3/2 EPR signal associated with native Fe-proteins of nitrogenase. *FEBS J.* **189**, 250-254 (1985).
10. P. A. Lindahl, E. P. Days, T. A. Kent, W. H. Orme-Johnson, E. Münck, Mössbauer, EPR, and Magnetization Studies of the *Azotobacter vinelandii* Fe Protein. *J. Biol. Chem.* **260**, 11160-11117 (1985).
11. W. H. Orme-Johnson *et al.*, Electron paramagnetic resonance of nitrogenase and nitrogenase components from *Clostridium pasteurianum* W5 and *Azotobacter vinelandii* OP. *Proc. Natl. Acad. Sci. U.S.A.* **69**, 3142-3145 (1972).
12. E. Jimenez-Vicente *et al.*, Sequential and differential interaction of assembly factors during nitrogenase MoFe protein maturation. *J. Biol. Chem.* **293**, 9812-9823 (2018).
13. A. Sosa Fajardo *et al.*, Structural insights into the mechanism of the radical SAM carbide synthase NifB, a key nitrogenase cofactor maturating enzyme. *J. Am. Chem. Soc.* **142**, 11006-11012 (2020).
14. A. W. Fay, J. A. Wiig, C. C. Lee, Y. Hu, Identification and characterization of functional homologs of nitrogenase cofactor biosynthesis protein NifB from methanogens. *Proc. Natl. Acad. Sci. U.S.A.* **112** (2015).
15. J. Wilcoxen *et al.*, Electron paramagnetic resonance characterization of three iron-sulfur clusters present in the nitrogenase cofactor maturase NifB from *Methanocaldococcus infernus*. *J. Am. Chem. Soc.* **138**, 7468-7471 (2016).
16. L. Curatti *et al.*, In vitro synthesis of the iron-molybdenum cofactor of nitrogenase from iron, sulfur, molybdenum, and homocitrate using purified proteins. *Proc. Natl. Acad. Sci. U.S.A.* **104**, 17626-17631 (2007).
17. D. Zhao, L. Curatti, L. M. Rubio, Evidence for *nifU* and *nifS* participation in the biosynthesis of the iron-molybdenum cofactor of nitrogenase. *J. Biol. Chem.* **282**, 37016-37025 (2007).

18. X. X. Li, Q. Liu, X. M. Liu, H. W. Shi, S. F. Chen, Using synthetic biology to increase nitrogenase activity. *Microb. Cell Fact.* **15**, 43 (2016).

19. E. V. Koskela, A. D. Frey, Homologous recombinatorial cloning without the creation of single-stranded ends: exonuclease and ligation-independent cloning (ELIC). *Mol. Biotechnol.* **57**, 233-240 (2015).

20. J. C. Crack, J. Green, A. J. Thomson, N. E. Le Brun, "Techniques for the production, isolation, and analysis of iron–sulfur proteins" in Metalloproteins. Methods in Molecular Biology, J. Fontecilla-Camps, Y. Nicolet, Eds. (Humana Press, Totowa, NJ, 2014), vol. 1122, pp. 33-48.

21. B. J. Hoffman *et al.*, Lactose fed-batch overexpression of recombinant metalloproteins in *Escherichia coli* BL21 (DE3): process, control yielding high levels of metal-incorporated, soluble protein. *Protein Exp. Purif.* **6**, 646-654 (1995).

22. E. Jimenez-Vicente *et al.*, Application of affinity purification methods for analysis of the nitrogenase system from *Azotobacter vinelandii*. *Methods Enzymol.* **613**, 231-255 (2018).

23. D. T. Petasis, M. P. Hendrich, Quantitative Interpretation of Multifrequency Multimode EPR Spectra of Metal Containing Proteins, Enzymes, and Biomimetic Complexes. *Methods Enzymol.* **563**, 171-208 (2015).

24. H. D. Hill, J. G. Straka, Protein determination using bicinchoninic acid in the presence of sulfhydryl reagents. *Anal. Biochem.* **170**, 203-208 (1988).

25. M. L. Moss, M. G. Mellon, Colorimetric determination of iron with 2,2'-bipyridyl and with 2,2',2'-terpyridyl. *Ind. Eng. Chem., Anal. Ed.* **14**, 862-865 (1942).

26. U. K. Laemmli, Cleavage of structural proteins during the assembly of the head of bacteriophage T4. *Nature* **227**, 680-685 (1970).

27. X. Jiang *et al.*, Exploiting genetic diversity and gene synthesis to identify superior nitrogenase NifH protein variants to engineer N_2-fixation in plants. *Commun. Biol.* **4**, 1-11 (2021).

28. P. C. Dos Santos, Molecular biology and genetic engineering in nitrogen fixation. *Methods Mol. Biol.* **766**, 81-92 (2011).

29. G. W. Strandberg, P. W. Wilson, Formation of the nitrogen-fixing enzyme system in *Azotobacter vinelandii*. *Can J. Microbiol.* **14**, 25-31 (1968).

30. V. K. Shah, J. R. Allen, N. J. Spangler, P. W. Ludden, In vitro synthesis of the iron-molybdenum cofactor of nitrogenase. Purification and characterization of NifB cofactor, the product of NIFB protein. *J. Biol. Chem.* **269**, 1154-1158 (1994).

Supporting Figures

A

```
AvNifD    MTGMSREEVESLIQEVLEVYPEKARKDRNKHLAVNDPAVTQS-----KKCIISNKKSQPG   55
RsNifD    ----------------MQFKCNQTLPERAIHIALKGPGGKCQRGDGTTCFIANNVATTPG   44
AvVnfD    -------------MPMVLLECDKDIPERQKHIYLKAPNEDTR-----EFLPIANAATIPG   42
AvAnfD    -------------MPHHEFECSKVIPERKKHAVIKGKGETLA-----DALPQGYLNTIPG   42
                            .  .:   :*  *  ::                     : **

                      65  69                         96
AvNifD    LMTIRGCAYAGSKGVVWGPIKDDIHISHGPVGCGQYSRAGRRNYYIGTTGVNAFVTMNFT   115
RsNifD    DMTERGCTYAGCRGVVGGPVKDAIQLTHGPIGCAFFSWGYRPHL-AD---SDFHMKYTFV   100
AvVnfD    TLSERGCAFCGAKLVIGGVLKDTIQMIHGPLGCAYDTWHTKRYP-TD--NGHFNMKYVWS   99
AvAnfD    SISERGCAYCGAKLVIGTPMKDVIHISHGPVGCTYDTWQTKRYI-SD--NDNFQLKYTYA   99
           :: ***::.*.: *:   :** *:: ***:**   :   :     .    .  :.  :

AvNifD    SDFQEKDIVFGGDKKLAKLIDEVETLFPLNKGISVQSECPIGLIGDDIESVSKVKGAE-L   174
RsNifD    TDMNETNIVFGGEKKLLQSIIEANAEFPNAKAVFVYNTCSTALIGDDGRDVAKQAEAI-I   159
AvVnfD    TDMKESHVVFGGEKRLEKSMHEAFDEMPDIKRMIVYTTCPTALIGDDIKAVAKKVMKDRP   159
AvAnfD    TDVKEKHIVFGAEKLLKQNIIEAFKAFPQIKRMTIYQTCATALIGDDINAIAEEVMEEMP   159
          :*.:*..:***.:* * : : *.   :*  * : :   *  .***** . :::

AvNifD    SKTIVPVRCEGFRGVSQSLGHHIANDAVRDWVLGKRDEDTTFASTPYDVAIIGDYNIGGD   234
RsNifD    GKPVVFFECEGFRGVSQSMGHHVGNETIFRQLVGSVEPEGD---FSRSINIIGDYNIKND   216
AvVnfD    DVDVFTVECPGFSGVSQSKGHHVLNIGWINEKVETMEKEIT---SEYTMNFIGDFNIQGD   216
AvAnfD    EVDIFVCNSPGFAGPSQSGGHHKINIAWINQKVGTVEPEIT---GDHVINYVGEYNIQGD   216
             :.  .. ** * *** ***  *       : . : :         :  :*::** .*

                                                  275
AvNifD    AWSSRILLEEMGLRCVAQWSGDGSISEIELTPKVKLNLVHCYRSMNYISRHMEEKYGIPW   294
RsNifD    IRTFEYLFEALGLRIIARFTGNVSVDDLKIMHKAALNIVHCQRSATYIADMMKDKYGTPY   276
AvVnfD    TQLLQTYWDRLGIQVVAHFTGNGTYDDLRCMHQAQLNVVNCARSSGYIANELKKRYGIPR   276
AvAnfD    QEVMVDYFKRMGIQVLSTFTGNGSYDGLRAMHRAHLNVLECARSAEYICNELRVRYGIPR   276
                  . :*:: :: ::*: : . :.   :. **::.* **  **.  :. :** *

AvNifD    MEYNFFGPTKTIESLRAIAAKFDESIQKKCEEVIAKYKPEWEAVVAKYRPRLEGKRVMLY   354
RsNifD    INVTLWGMKNMAKALRDTAAFFG--LEARAEEVIAREVARIQPYIDAYRQRLQGKRVFIY   334
AvVnfD    LDIDSWGFNYMAEGIRKICAFFG--IEEKGEELIAEEYAKWKPKLDWYKERLQGKKMAIW   334
AvAnfD    LDIDGFGFKPLADSLRKIGMFFG--IEDRAKAIIDEEVARWKPELDWYKERLMGKKVCLW   334
          ::   :* .   ..:*     *.  :: : : :* .   . :  :  *: ** **:: ::

              360                   380
AvNifD    IGGLRPRHVIGAY-EDLGMEVVGTGYEFAHNDDYDRTMKEMGDSTLLYDDVTGYEFEEFV   413
RsNifD    QGGPRVWHWIELL-RELGMETETAATTFGHTDDYEKIFNQIGEGALVIDNPNVPEIEEIL   393
AvVnfD    TGGPRLWHWTKSVEDDLGVQVVAMSSKFGHEEDFEKVIARGKEGTYYIDDGNELEFFEII   394
AvAnfD    PGGSKLWHWAHVIEEEMGLKVVSVYIKFGHQGDMEKGIARCGEGTLAIDDPNELEGLEAL   394
           ** :  *       ::*::.      *.*  * :: : .  :.:   *: .  *  * :

                       426               440 442
AvNifD    KRIKPDLIGSGIKEKFIFQKMGIPFREMHSWDYSGPYHGFDGFAIFARDMDMTLNNPCWK   473
RsNifD    TRRRPDLFISGNKERYLAYKMGVPFVNGHTYD-TGPYAGFVGMVNFARDIDKALHAPVWN   452
AvVnfD    DLVKPDVIFTGPRVGELVKKLHIPYVNGHGYH-NGPYMGFEGFVNLARDMYNAVHNPLRH   453
AvAnfD    EMLKPDIILTGKRPGEVAKKVRVPYLNAHAYH-NGPYKGFEGWVRFARDIYNAIYSPIHQ   453
             :**:: :* :   :  *: :*: : * :. .*** ** * . :***:  ::  *  :

AvNifD    KLQAPWEASEGAE--------KVAASA---------------------------------   492
RsNifD    IVHQHARPAPVAR-----------------HAVHGSEEVES-------------------   476
AvVnfD    LAAVDIRDKSQTT--------PVIVRGAA-------------------------------   474
AvAnfD    LSGIDITKDNAPEWGNGFRTRQMLSDGNLSDAVRNSETLRQYTGGYDSVSKLREREYPAF   513

AvNifD    -----     492
RsNifD    -----     476
AvVnfD    -----     474
AvAnfD    ERKVG     518
```

B

```
AvNifD          MSQQVDKIKASYPLFLDQDYKDMLAKKRDGFEEKYPQDKIDEVFQWTTTKEYQELNFQRE   60
RsNifD          --------------------------------------------------MTSCLTLQER   10
AvVnfD          ---------------------------------------MSNCELTVLKPAEVKLSPRDR   21
AvAnfD          --------------------------------------------------MTCEVKEKGR   10
                                                                     :. : .

AvNifD          ALTVNPAKACQPLGAVLCALGFEKTMPYVHGSQGCVAYFRSYFNRHFREPVSCVSDSMTE   120
RsNifD          AVAINPTRSCAPIGAMLANYGIHGAITINHGSQGCATYPRHQMSRHFREPVEVATTSLTE   70
VnfK            EGIINPMYDCQPAGAQYAGIGIKDCIPLVHGGQGCTMFVRLLFAQHFKENFDVASTSLHE   81
AvAnfD          VGTINPIFTCQPAGAQFVSIGIKDCIGIVHGGQGCVMFVRLIFSQHYKESFELASSSLHE   70
                   :**    *  * **     *:.  :    **.***. : *   : :*::* .. .: *: *

AvNifD          DAAVFGGQQNMKDGLQNCKATYKP-DMIAVSTTCMAEVIGDDLNAFINNSKK----EGFI   175
RsNifD          KTTVYGGKQNLLAALKNIWERFHP-TMIMVCSTCLSETIGDDIPAIIDE-----FLDKHP   124
AvVnfD          ESAVFGGAKRVEEGVLVLARRYPNLRVIPIITTCSTEVIGDDIEGSIRVCNRA-LEAEFP   140
AvAnfD          DGAVFGACGRVEEAVDVLLSRYPDVKVVPIITTCSTEIIGDDVDGVIKKLNEGLLKEKFP   130
                . :*:*.   .:   .:         :    :: : :** :* ****: .  *          .

AvNifD          PDEFPVPFAHTPSFVGSHVTGWDNMFEGIARYFTLKSMDDKVVGSNKKINIVPGFETYLG   235
RsNifD          DVTIPILSVKTPSYIGNHTTGFDNFLKEIALNLPDR--RKKKGETNGRINIIPGWVN-PG   181
AvVnfD          DRKIYLAPVHTPSFKGSHVTGYAECVKSVFKTITDA---HGKGQPSGKLNVFPGWVN-PG   196
AvAnfD          DREVHLIAMHTPSFVGSMISGYDVAVRDVVRHFA------KREAPNDKINLLTGWVN-PG   183
                  . :     :***: *.  :*:    .. :    :             . ::*:. *: .  *

AvNifD          NFRVIKRMLSEMGVGYSLLSDPEEVLDTPAD-GQFRMYAGGTTQEEMKDAPNALNTVLLQ   294
RsNifD          DIRELKHMLREMGLHGLWITDYSETLDGGYYDPRPHVPRGGTTIEELRSSSKSLATIALQ   241
AvVnfD          DVVLLKRYFKEMDVEANIYMD-TEDFDSPMLPNKSIETHGRTTVEDIADSANALATLSLA   255
AvAnfD          DVKELKHLLGEMDIEANVLFE-IESFDSPILPDGSAVSHGNTTIEDLIDTGNARATFALN   242
                :.   :*: : **.:        :  * :*              * ** *:: .: ::   *. *

AvNifD          PWHLEKTKKFVEGTWKHEVPKLNIPMGLDWTDEFLMKVSEISGQPIPASLTKERGRLVDM   354
RsNifD          RHVGGEAARIYERRYNVPAHVLTMPIGLKNTDAFVNTLIEITDHTIPESLEVERARLLDA   301
AvVnfD          RYEGNTTGELLQKTFAVPNALVNTPYGIKNTDDMLRKIAEVTGKEIPESLVRERGIALDA   315
AvAnfD          RYEGTKAAEYLQKKFEIPAIIGPTPIGIRNTDIFLQNLKKATGKPIPQSLAHERGVAIDA   302
                      : .   :   :              * *:   **  :: .: : :.:  ** **   **.  :*

AvNifD          MTD-SHTWLHGKRFALWGDPDFVMGLVKFLLELGCEPVHILCHNGNKR---WKKAVDAIL   410
RsNifD          LVD-THMYTTGLRVALYGDPDLLEGLVGLIAEMGMTPAYILTAADNRP---WGERMVELT   357
AvVnfD          LADLAHMFFANKKVAIFGHPDLVLGLAQFCMEVELEPVLLLIGDDQGNKYKKDPRIEELK   375
AvAnfD          LADLTHMFLAEKRVAIYGAPDLVIGLAEFCLDLEMKPVLLLLGDDN-SKYVDDPRIKALQ   361
                :.* :* :     :.*::* **:: **. :   ::    *. :*     .:             :   :

AvNifD          AASPYGKNATVYIGKDLWHLRSLVFTD--KPDFMIGNSYGKFIQRDTLHKGKEFEVPLIR   468
RsNifD          GE--LGVESEIILKGDLHELHKRIKQQ--PVDLLIGHSKGRFIAE-------AENIPLVR   406
AvVnfD          NT--AHFDIEIVHNADLWELEKRI-NAGLQLDLIMGHSKGRYVAI-------EANIPMVR   425
AvAnfD          EN--VDYGMEIVTNADFWELENRIKNEGLELDLILGHSKGRFISI-------DYNIPMLR   412
                            :     *: .*.. :         *:::*:* *:::             ::*::*

AvNifD          IGFPIFDRHHLHRSTTLGYEGAMQILTTLVNSILERLDEETRGMQATDYNHD-------L   521
RsNifD          VGFPVEDRFGHHRRSIVGYNGAIALVDEITNTIFERRATTIVSNTLIETGVEGPTSVPIA   466
AvVnfD          VGFPTFDRAGLYRKPSIGYQGAMELGEMIANAMFAHMEYTRNKEWILNTW----------   475
AvAnfD          VGFPTYDRAGLFRYPTVGYGGAIWLAEQMANTLFADMEHKKNKEWVLNVW----------   462
                :***   **    .*    :** **: :    :.*:::                :

AvNifD          VR------- 523
RsNifD          LRNGTTAHG 475
AvVnfD          --------- 475
AvAnfD          --------- 462
```

Figure S1: Amino acid sequence alignment of NifDK^{RS} with the corresponding Nif, Vnf and Anf polypeptides of *A. vinelandii*. Alignments were produced with Clustal Omega. **(A)** NifD alignment using *A. vinelandii* NifD (P07328), VnfD (P16855), AnfD (P16266), and

Roseiflexus NifD (A5USK3). (**B**) NifK alignment using *A. vinelandii* NifK (P07329), VnfK (C1DI23), AnfK (P16267), and Roseiflexus NifK (A5USK2). Amino acid numbering corresponds to the *A. vinelandii* NifD. Conserved residues are highlighted in green. Black boxes mark amino acid residues that serve as FeMo-co ligands and others that are conserved in the FeMo-co environment. Blue boxes mark residues involved in P-cluster coordination and environment in D and K polypeptides. Amino acid residues unique to V and Fe-only nitrogenases are highlighted in red. Green arrows point to NifD^{RS} residues that suggest a Mo nitrogenase, either because they are unique to Mo nitrogenases or because they differ from VnfD and AnfD unique residues. The red arrow points to a residue that does not follow this rule. Black arrows point to FeMo-co coordinating residues.

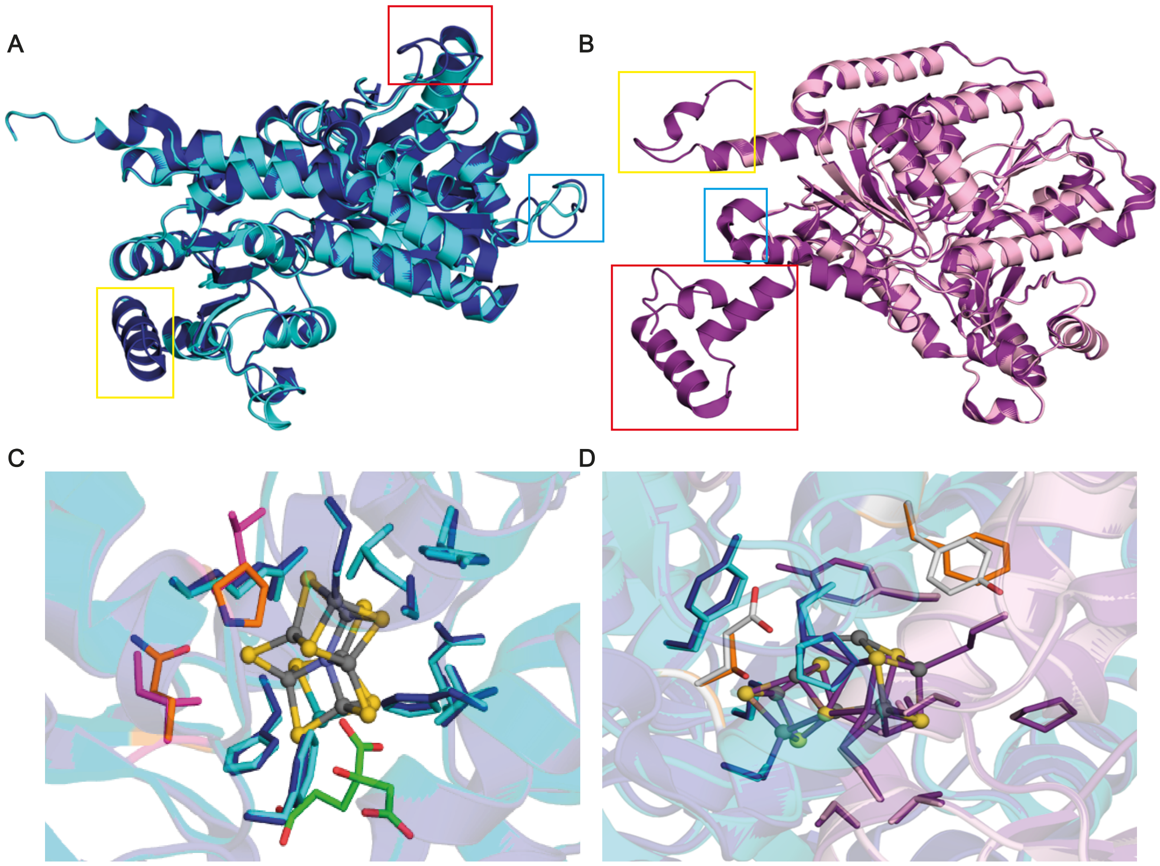

Figure S2. Overlay of NifDK^{RS} and NifDK^{Av} structures and comparison of the FeMo-co and P-cluster environments. A ProMod3 model of NifDK^{RS} and the crystal structure of NifDK^{Av} (PDB ID: 3U7Q) were used. **A)** Overlay of NifD subunits showing NifD^{Av} in dark blue and NifD^{RS} in cyan. Color squares mark structural differences: yellow for the 4-19 residue α-helix absent in NifD^{RS}, red and blue for differences in the 101-108 and the 208-214 residue loop, respectively. **B)** Overlay of NifK subunits showing NifK^{Av} in purple and NifK^{RS} in pink. Color squares mark structural features that were absent in NifK^{RS}: red for the 2-56 residue segment, blue for the 453-462 residue loop and α-helix, and yellow for the 509-534 segment not modeled in NifK^{RS}. **C)** Environment at 4 Å around FeMo-co. Conserved amino acid residues in NifD^{Av} (Val^{70}, Arg^{96}, His^{195}, Tyr^{229}, Ile^{231}, Cys^{275}, Ser^{278}, Gly^{356}, Gly^{357}, Arg^{359}, Phe^{381}, His^{442}) and NifD^{RS} (Val^{59}, Arg^{85}, His^{180}, Tyr^{211}, Ile^{213}, Cys^{257}, Ser^{260}, Gly^{336}, Gly^{337}, Arg^{339}, Phe^{361}, His^{422}) are shown in dark blue and cyan, respectively, while non-conserved residues in NifD^{Av} (Ile^{355}, Leu^{358}) and NifD^{RS} (Gln^{335}, Pro^{338}) are shown in magenta and orange, respectively. The inorganic moiety of FeMo-co is shown in ball and sticks while homocitrate is shown in sticks. FeMo-co atom color code: S in yellow, Fe in grey, Mo in cyan, C in green, O in red. **D)** Environment at 4 Å around the P-cluster. The color codes of conserved amino acid residues, P-cluster atoms, and ribbon structures are the same as in previous panels. Conserved

amino acid residues are shown for NifD^{Av} (Cys^{62}, Tyr^{64}, Pro^{85}, Gly^{87}, Cys^{88}, Cys^{154}, Gly^{185}), NifD^{RS} (Cys^{51}, Tyr^{53}, Pro^{74}, Gly^{76}, Cys^{77}, Cys^{139}, Gly^{170}), NifK^{Av} (Cys^{70}, Pro^{72}, Ser^{92}, Gly^{94}, Cys^{95}, Tyr^{198}, Thr^{152}, Cys^{153}, Ser^{188}), and NifK^{RS} (Cys^{20}, Pro^{22}, Ser^{42}, Gly^{44}, Cys^{45}, Tyr^{48}, Thr^{102}, Cys^{103}, Ser^{137}). Non conserved amino acid residues in NifD^{Av} (Tyr^{91}, Glu^{153}) and NifD^{RS} (Phe^{80}, Thr^{138}) are shown in white and orange, respectively.

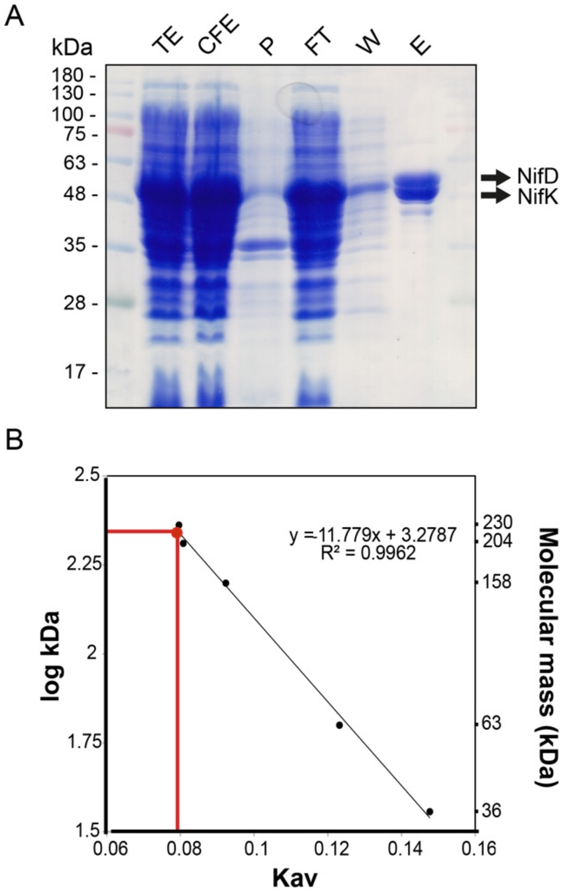

Figure S3. Purification of NifDK^{RS} from recombinant *E. coli* cells. A) SDS-PAGE analysis of the NifDK^{RS} purification process. Purification fractions were loaded on 12% denaturing acrylamide gels in the following order: Total Extract (TE), Cell-Free Extract (CFE), Pellet or insoluble fraction (P), Flow Trough (FT), Wash (W) and Elution (E). **B)** NifDK^{RS} molecular mass determination by size-exclusion chromatography. NifDK^{RS} migration is represented by a red dot. Protein standards are represented by black dots in decreasing order of mass: NifDK^{Av} (230 kDa), NifEN^{Av} (204 kDa), Aldolase (158 kDa), NifH^{Av} (63 kDa) and *M. infernus* NifB^{Mi} (36 kDa). Linear regression fit parameters are shown.

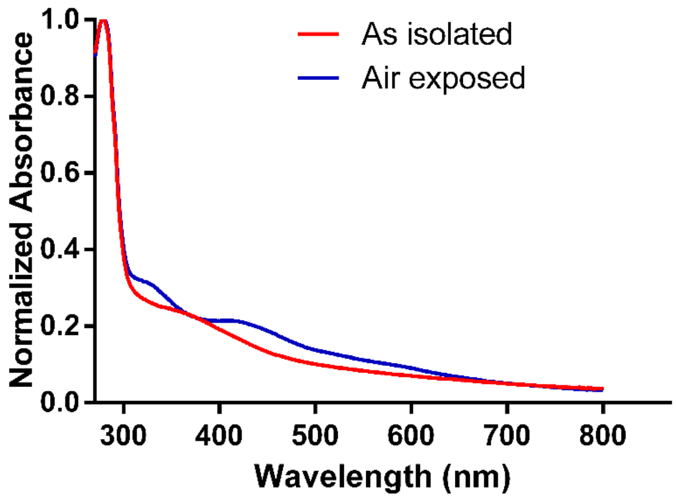

Figure S4. **UV-Visible spectra of purified apo-NifDK*RS* in as isolated and air-exposed conditions**. Apo-NifDK*RS* had been co-expressed with NifH*RS* and NifUS*Av*. The changes in the UV-visible spectrum are indicative of the sensitivity of its [FeS] clusters to O_2.

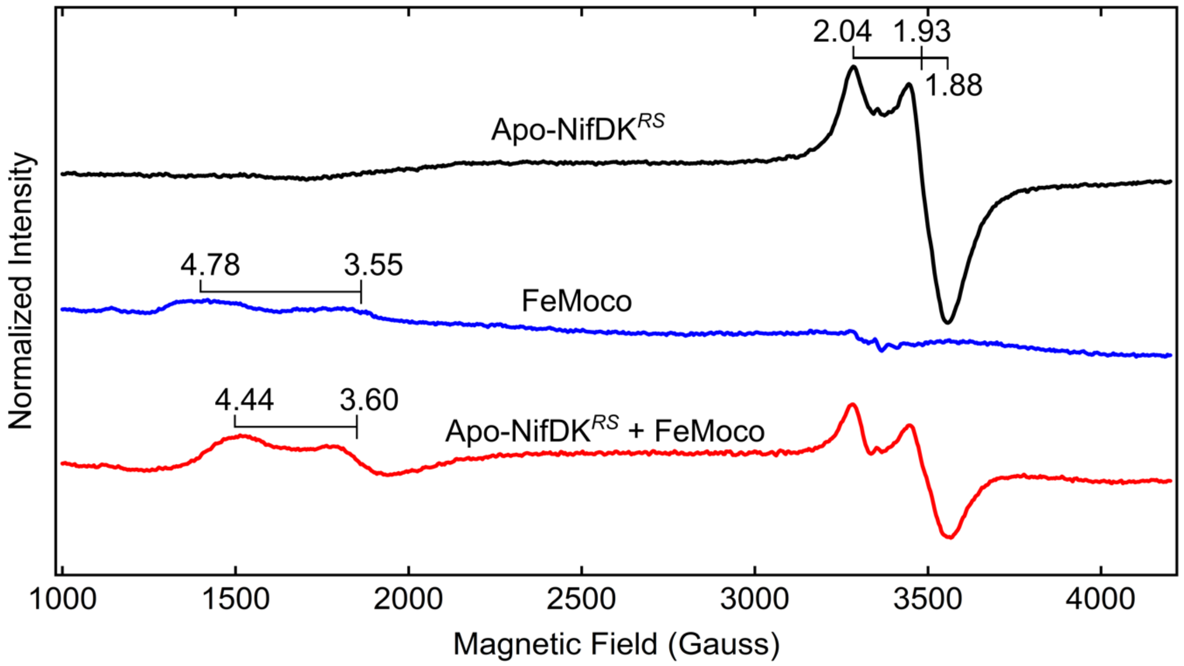

Figure S5. Effect of FeMo-co on apo-NifDK^{RS} EPR signals. Spectra of DTH-reduced apo-NifDK^{RS} (black trace), pure FeMo-co (blue trace), and a repurified apo-NifDK^{RS} after incubation with FeMo-co (red trace) are shown. Relevant EPR signal *g* values are shown. Spectra were recorded (10 scans) at microwave power of 20 mW and temperature of 12 K.

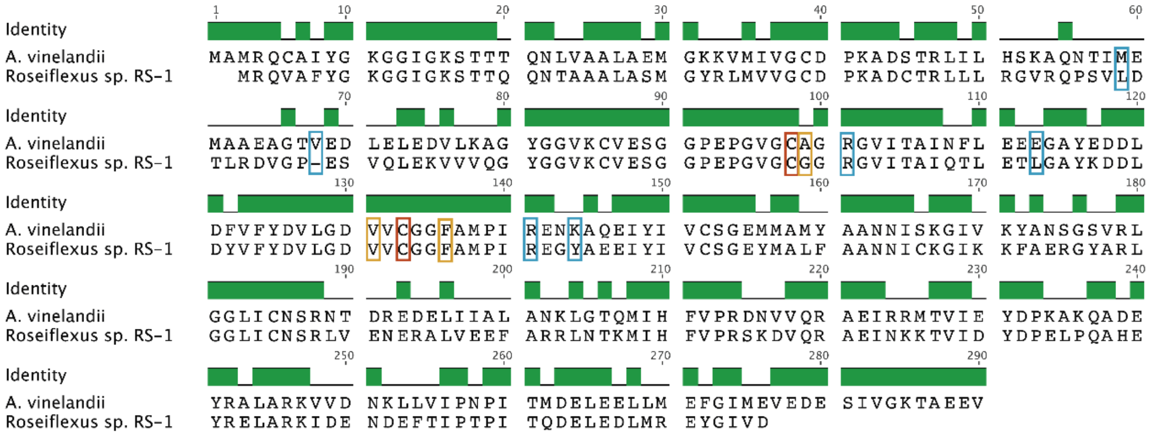

Figure S6. Amino acid sequence alignment of NifH from *A. vinelandii* (NifH^{Av}) and *Roseiflexus* sp. RS-1 (NifH^{RS}). The [Fe_4S_4] coordinating residues are shown inside red boxes; residues involved in NifDK interaction are shown in blue boxes; residues involved in other important interactions are shown in orange boxes. This alignment was obtained using Geneious version 7.1 (http://www.geneious.com).

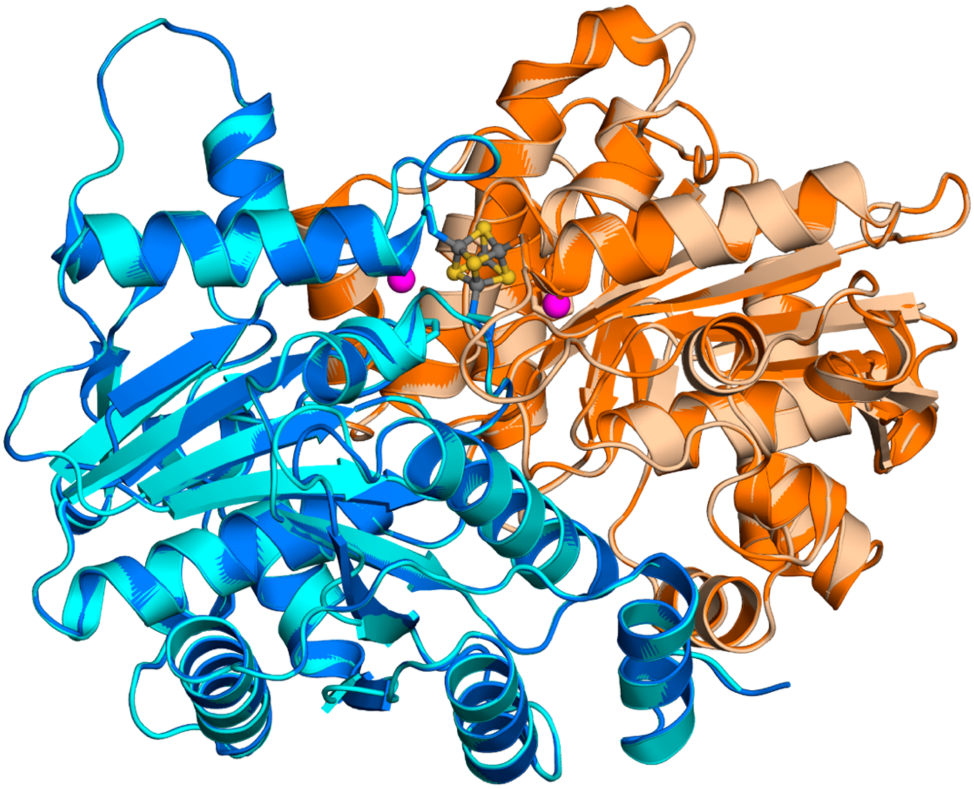

Figure S7. Modeled 3D structure of the NifH^{RS} dimer and overlap with the *M. acetivorans* NifH structure (NifH^{Ma}). Dark blue and orange correspond to the with NifH^{Ma} subunites (PDB: 6NZJ) while cyan and light orange correspond to the NifH^{RS} subunits. The pink spheres show the positions of A^{96} methyl groups in each chain of NifH^{Ma}. These groups are absent in NifH^{RS} which present G^{96} residues instead.

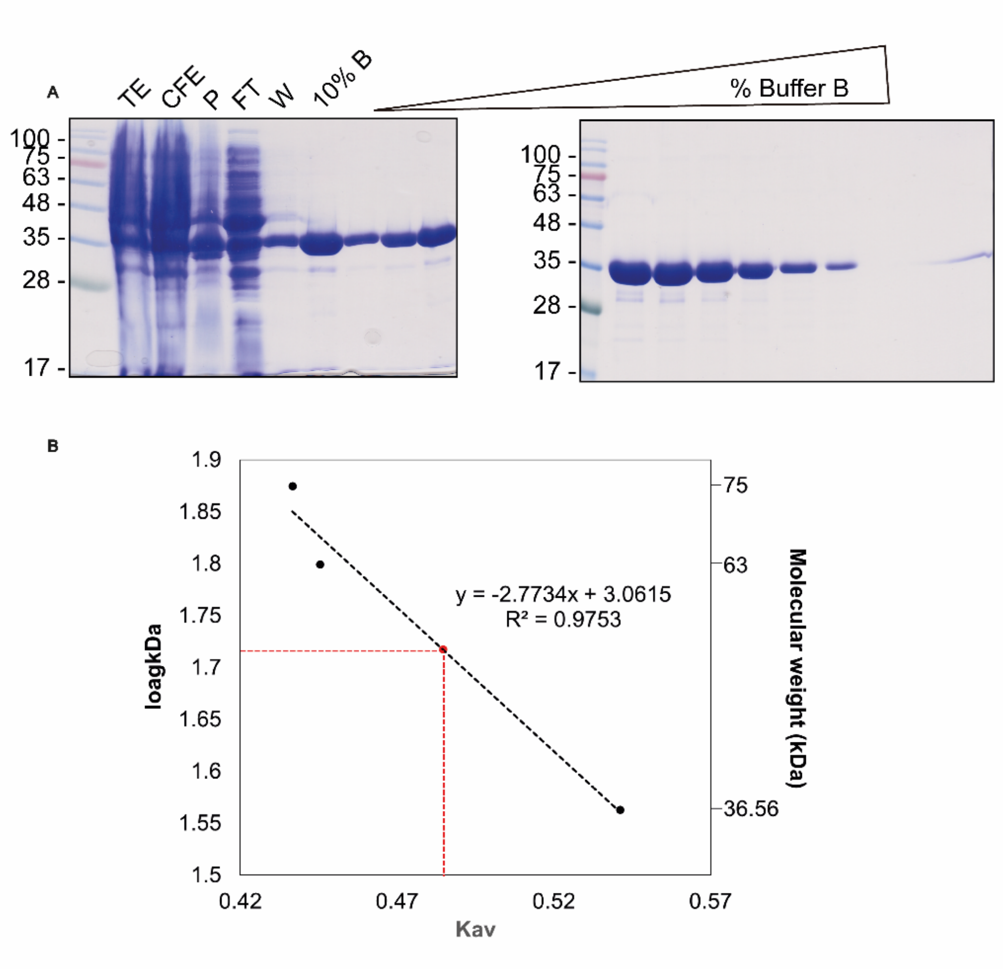

Figure S8. **NifH^{RS} isolation from recombinant *E. coli* cells. A)** SDS-PAGE analysis of the purification process. Purification fractions were loaded into SDS-PAGE gels in the following order: Total extract (TE), soluble cell free extract (CFE), insoluble pellet (P), chromatography flow through (FT), chromatography wash (W), second wash with 10% buffer B (10% B) and protein elution trough an imidazole gradient (% Buffer B). **B)** NifH^{RS} molecular mass determined by size-exclusion chromatography. NifH^{RS} migration is represented by a red dot. Protein standards are represented by black dots in decreasing order of mass including conoalbumin (75 kDa), NifH^{Av} (63 kDa) and NifB^{Mi} (36.5 kDa).

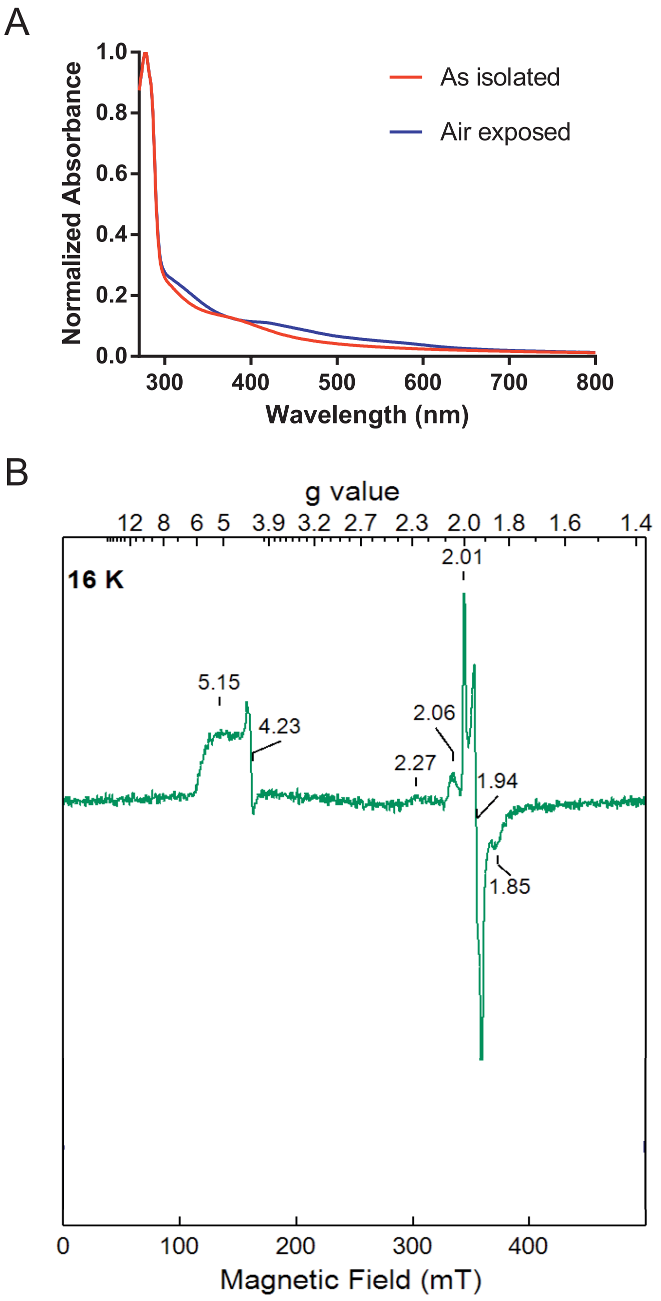

Figure S9. Spectroscopic characterization of NifH^{RS}. NifH^{RS} had been co-expressed with the *isc*^{Ec} gene cluster. **A)** UV-Visible spectra of as isolated (red line) and air-exposed NifH^{RS} (blue line). The changes in the UV-visible spectrum are indicative of the sensitivity of its [FeS] clusters to O_2. **B)** EPR spectrum of DTH-reduced NifH^{RS}. The most representative *g* values are indicated.

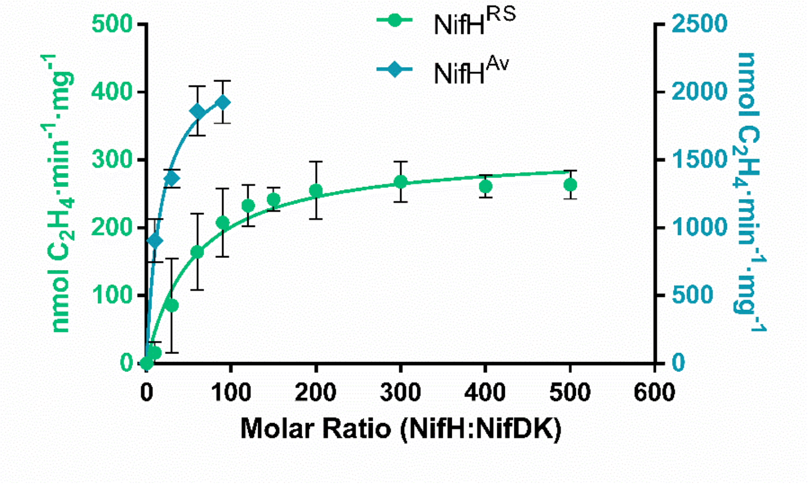

Figure S10. Titration of NifDK^{Av} activity with NifH^{RS} or NifH^{Av}. Reactions were carried out at 30 ºC for 15 minutes. Activities are expressed in nmol $C_2H_4 \cdot min^{-1} \cdot mg^{-1}$ of NifDK^{Av} protein. Data shown are average activities ± SD ($n \geq 2$).

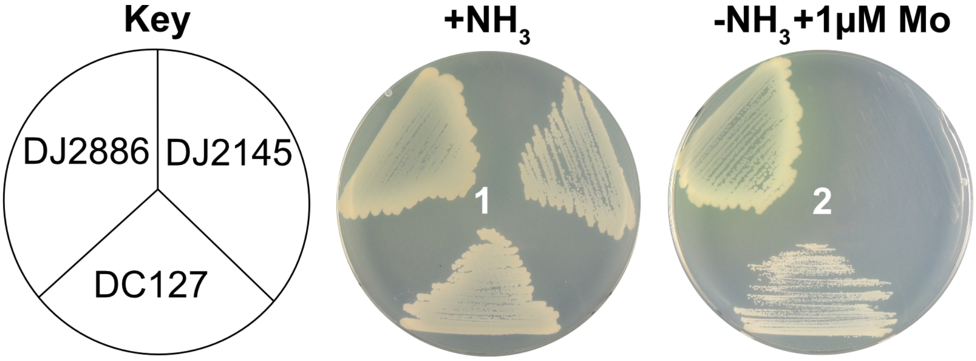

	Relevant genotype for:		
Strain	*nifH*	*vnfH*	alternative systems
DJ2886	*nifH*^{Av},		Δ*vnfDGK*::Sm^R, Δ*anfDGK*::Gen^R
DJ2145	Δ*nifH*,	Δ*vnfH*::Km^R	
DC127	*nifH*^{Rs},	Δ*vnfH*::Km^R,	Δ*vnfDGK*::Sm^R, Δ*anfDGK*::Gen^R

Figure S11. Phenotypic characterization of an *A. vinelandii* strain with *nifH*^{RS} replacing the native *nifH*^{Av}. Strains were cultured on Burk's medium agar plates containing a fixed nitrogen source (+NH$_3$) or under diazotrophic conditions (–NH$_3$ supplemented with 1 µM Mo). DC127 carries *nifH*^{RS} replacing *nifH*^{Av} at its native locus and additionally lacks *vnfH*, *vnfDGK*, and *anfDGK*. For comparison, DJ2886 (expressing only the Mo nitrogenase) and DJ2145 (with deleted *nifH* and *vnfH* genes) were also cultured. DC127 diazotrophic growth is slower than DJ2886. Relevant genotypes for each strain are indicated. DJ strains were kindly provided by Dennis Dean.

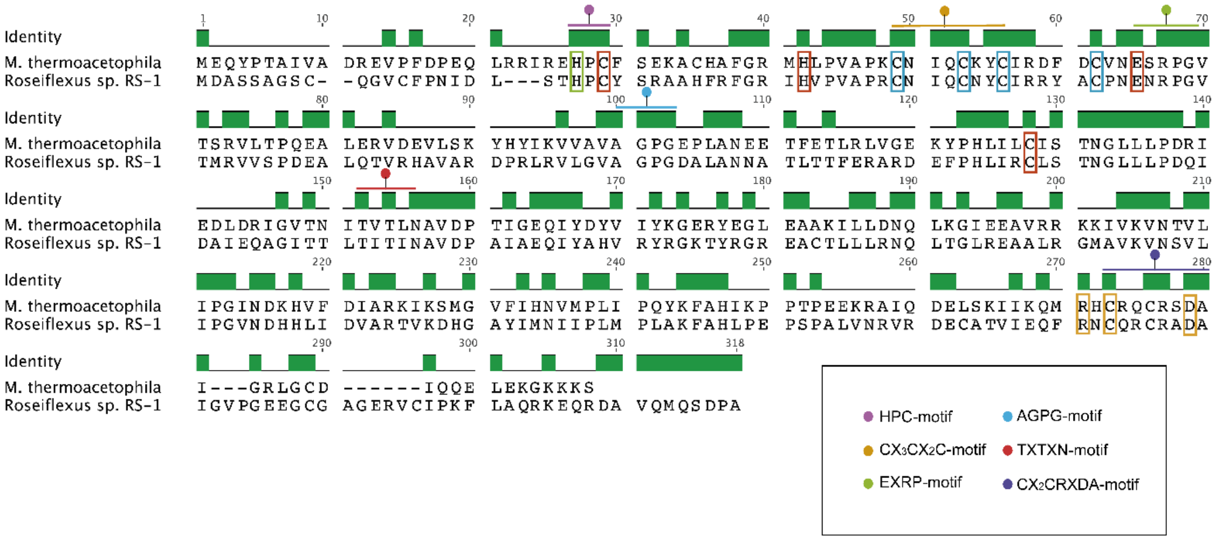

Figure S12. Amino acid sequence alignment of NifB from *M. thermoacetophila* (NifB^{Mt}) and *Roseiflexus* sp. RS-1 (NifB^{RS}). Residues involved in [Fe_4S_4] cluster coordination are shown in different colors: blue for RS-cluster ligands, red for K1-cluster and orange for K2-cluster ligands. The conserved histidine residue of the HPC-motif is framed by a green box. Conserved motifs are color labeled above the sequences following the legend. This alignment was obtained using Geneious version 7.1 (http://www.geneious.com).

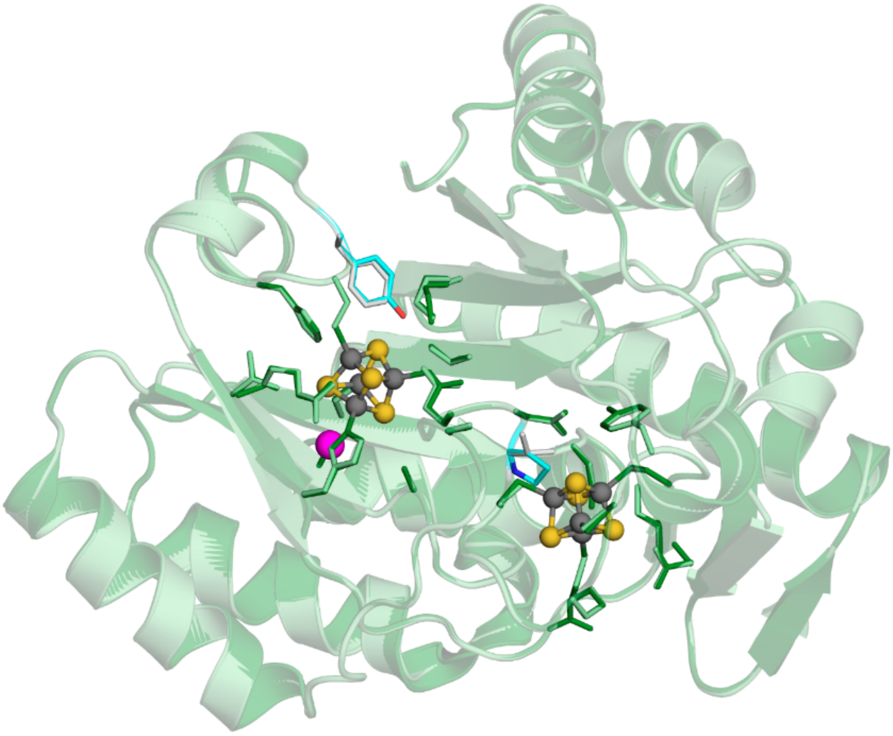

Figure S13. NifB^{RS} structural model constructed using NifB^{Mt} (6Y1X) as template. Overlap in which dark green corresponds to the 6Y1X NifB^{Mt} structure and light green corresponds to the modelled NifB^{RS} structure. Only the RS (left) and K1 (right) clusters are shown in the structure because K2 was no present in the NifB^{Mt} crystal structure. The magenta sphere marks the position of A^{98} in NifB^{Mt} that is replaced by G^{93} in NifB^{RS}. In addition, F^{30} and P^{58} in NifB^{Mt} (white sticks) change to T^{25} and V^{63}, respectively, in NifB^{RS} (cyan sticks).

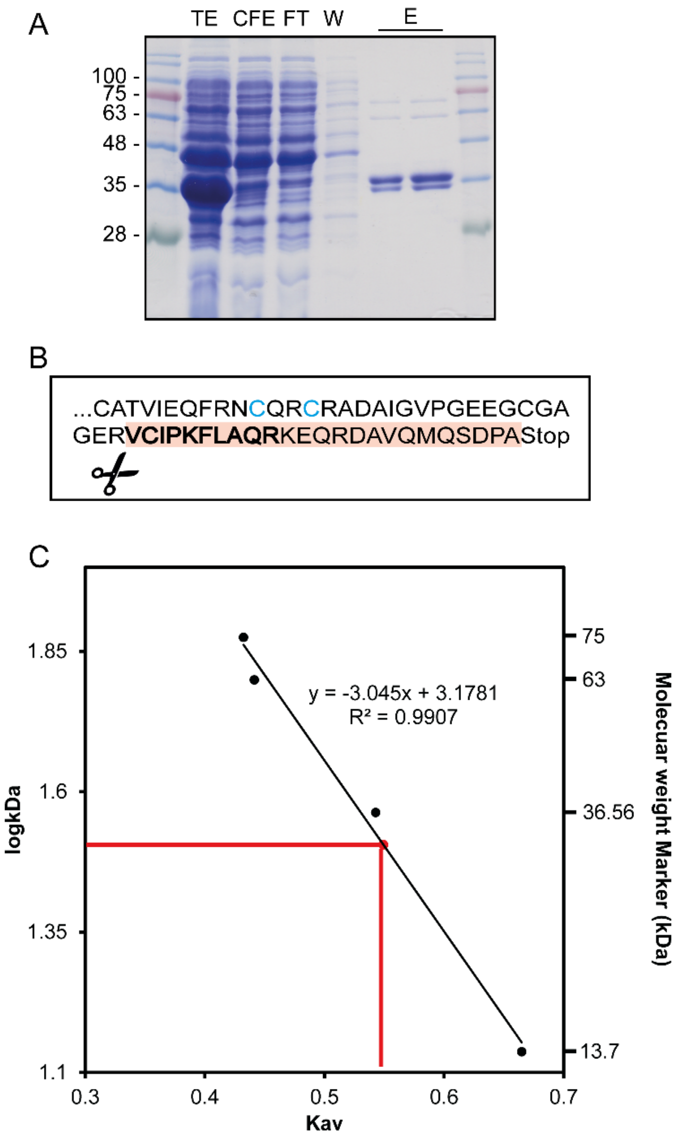

Figure S14. NifB^{RS} isolation from *E. coli* recombinant cells. A) SDS-PAGE analysis of a typical NifB^{RS} purification procedure based on StrepTactin affinity chromatography. Purification fractions were loaded into SDS-PAGE gels in the following order: Total extract (TE), cell-free soluble extract (CFE), column flow through (FT), wash (W), and elution fractions (E). **B)** C-terminal NifB^{RS} amino acid sequence with bold characters showing the MALDI detected end peptide. The light red background shows missing amino acids in the truncated NifB^{RS} version. The K2-cluster ligating cysteine residues (C^{268} and C^{271}) are shown in blue indicating their presence in the NifB^{RS} truncated form. **C)** NifB^{RS} molecular mass was determined using size-exclusion chromatography. NifB^{RS} migration is represented by a red dot. Proteins used as standards are represented by black dots in decreasing order of mass including cobalbumin (75 kDa), NifH^{Av} (63 kDa), NifB^{Mi} (36 kDa) and ribonuclease (13.7 kDa).

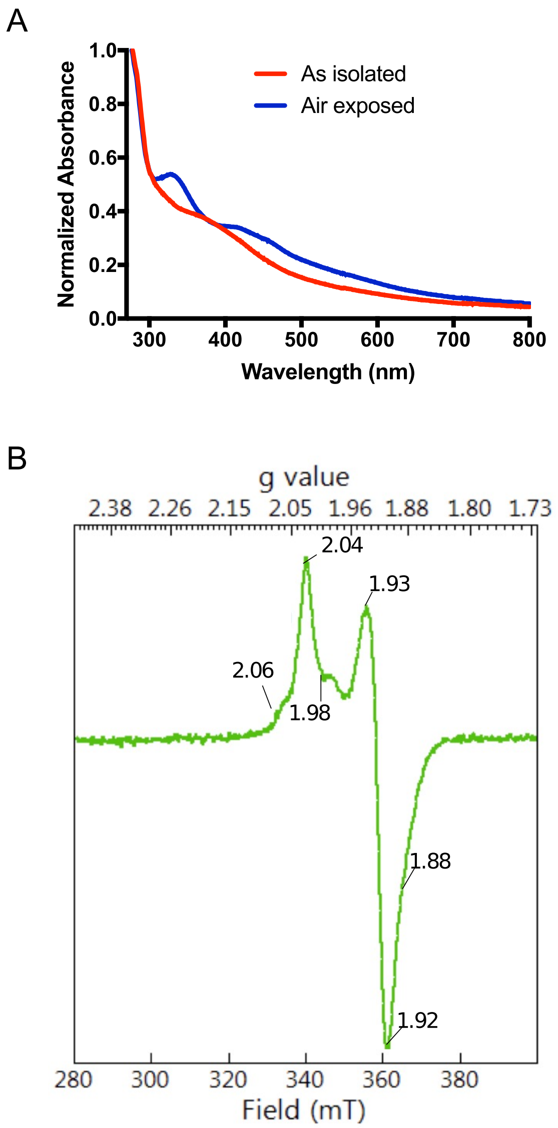

Figure S15. Spectroscopic characterization of NifB*RS*. NifB*RS* had been co-expressed with NifUS*Av*. **A)** UV-Visible spectra of *as isolated* (red line) and air-exposed NifB*RS* (blue line). The changes in the UV-visible spectrum are indicative of the sensitivity of its [FeS] clusters to O_2. **B)** EPR signal of DTH-reduced NifB*RS*. Representative *g* values corresponding to the three [Fe_4S_4] clusters of NifB proteins are indicated.

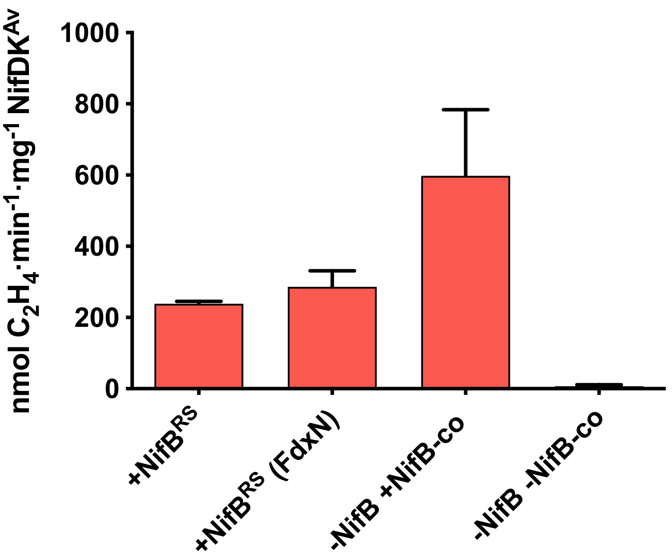

Figure S16. NifB^{RS} functions in the *in vitro* synthesis of FeMo-co. NifB^{RS} was obtained from recombinant *E. coli* cells co-expressing *nifU*^{Av}, *nifS*^{Av} and, when indicated, *fdxN*^{Av} genes. Reactions lacking NifB^{RS} were used as negative control whereas reactions including NifB-co (the product of NifB) in place of NifB^{RS} were used as positive controls. The activities of reconstituted NifDK^{Av} are average values obtained ± SD (n=2).

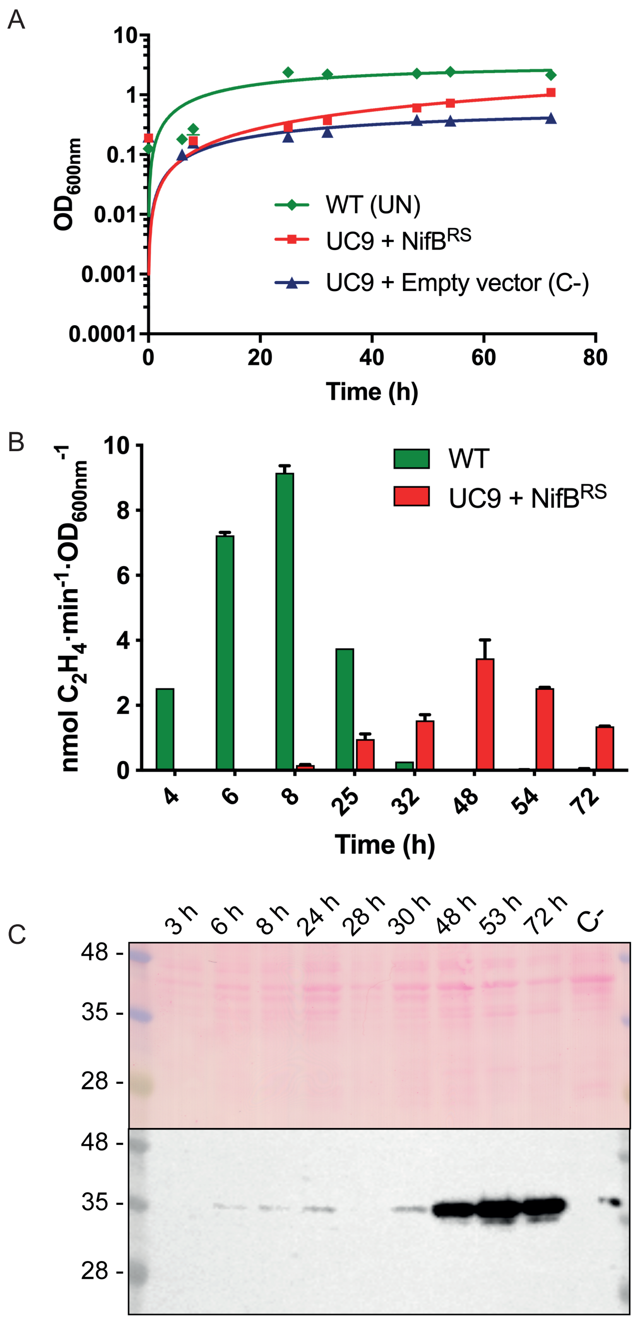

Figure S17. ***In vivo*** **genetic complementation of** ***nifB*** **deletion in** ***K. oxytoca*** **UC9 by** ***nifB^{RS}*****.** **A)** Diazotrophic growth of *K. oxytoca* wild type (green) and the Δ*nifB* UC9 strain transformed with pN2LP41, an expression plasmid carrying *his-nifB^{RS}* (red). Empty vector C- refers to UC9 transformed with pN2SB73, the expression plasmid lacking *nifB^{RS}* **B)** Time course of *in vivo* acetylene reduction. Data shown is the average of at least two replicates ± SD. **C)** His-NifB^{RS} detection using α-His antibody following nitrogenase derepression and simultaneous IPTG induction. The C- lane corresponds to UC9 transformed with pN2SB73 at 72 h of derepression.

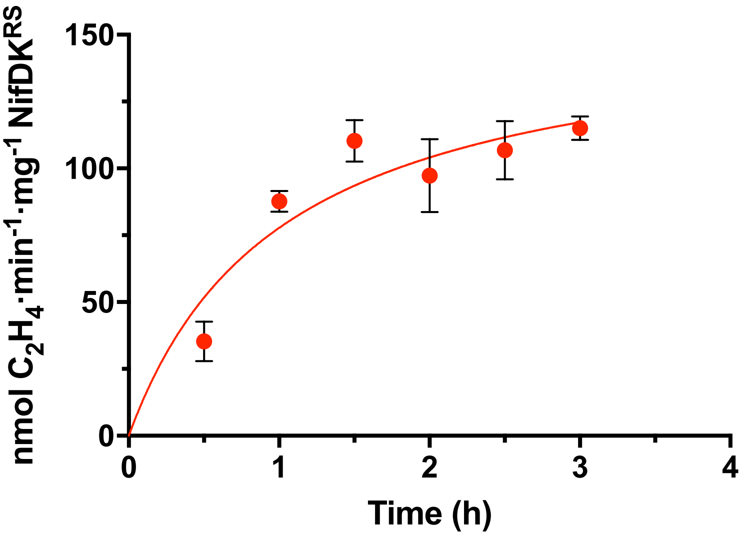

Figure S18. **Time course of FeMo-co synthesis and NifDK^{RS} reconstitution**. Incubation times during the FeMo-co synthesis phase were 0.5, 1, 1.5, 2, 2.5 and 3 h. All assays were carried out at 48 ºC, in the presence of Mo, and contained NifB^{RS}, NifX^{Av}, NifH^{RS}, and apo-NifDK^{RS}. Negative control assays lacking NifB^{RS} showed negligible activity. Data represents average activity ± SD (n=2).

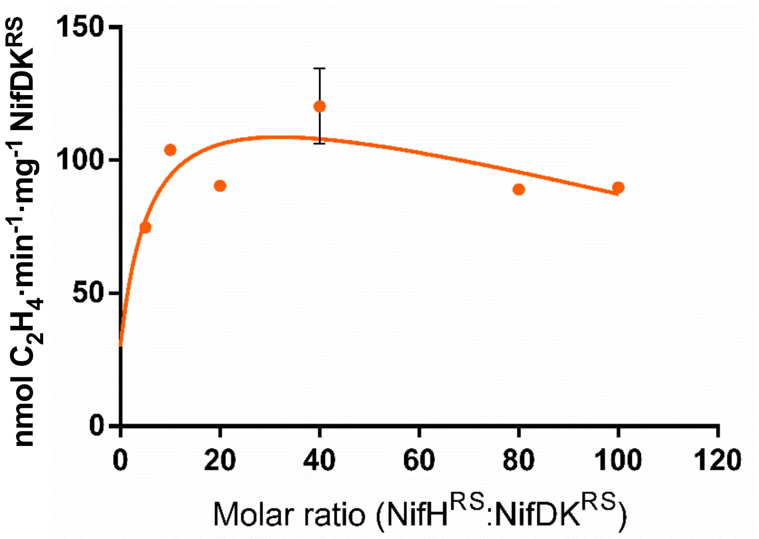

Figure S19. **Titration of NifDK^{RS} activity with NifH^{RS}.** NifB^{RS}-dependent NifDK^{RS} reconstitution reactions were performed for 90 minutes at 48 ºC. The NifDK^{RS} to NifH^{RS} molar ratios used in the acetylene reduction assay were 1:5, 1:10, 1:20, 1:40, 1:80 and 1:100. Negative control reactions lacking NifB^{RS} yielded 4.2 ± 1.7 C_2H_4·min^{-1}·mg^{-1} NifDK^{RS}. Data are average activities ± SD (n=2).

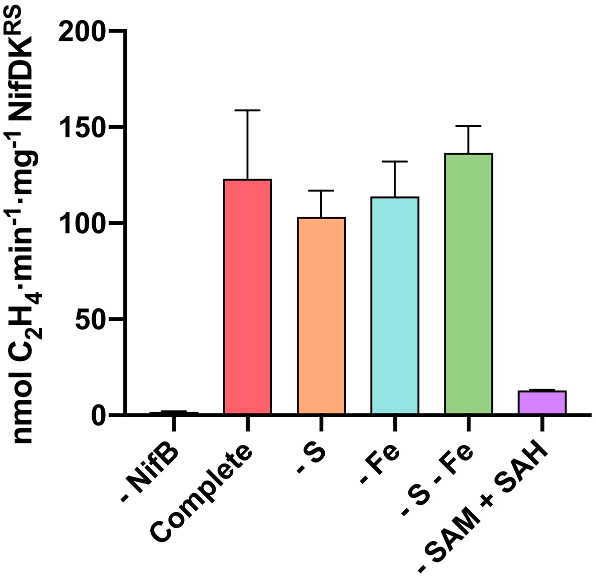

Figure S20. Sulfur, iron, and SAM dependency of NifB*^RS^*-dependent FeMo-co synthesis and insertion into apo-NifDK*^RS^*. Protein components of the complete reactions are described in Supporting Materials and Methods. The complete reactions contained Mo, Fe, S, SAM, and homocitrate as substrates. Data represents average activities ± SD (n=2).

Supporting Tables

Table S1. Conserved amino acid residues in the FeMo-co and P-cluster environments of NifDK^{Av} and NifDK^{RS}.

**FeMo-co coordination**		**P-cluster coordination**	
**NifDK^{Av} residue**	**NifDK^{RS} residue**	**NifDK^{Av} residue**	**NifDK^{RS} residue**
C275	C257	C62	C51
H442	H422	C88	C77
S278	S260	C154	C139
G356	G336	S92	S81
G357	G337	C70 (β)	C20 (β)
R96	R85	C95 (β)	C45 (β)
R359	R339	C153 (β)	C103 (β)
H195	H180	S188 (β)	C137 (β)
G69	G58	G94 (β)	G44 (β)
V70	V59	G87	G76
Q191	Q176	G185	G170
E427	E407		

Residues located at NifK are indicated as (β). All other amino acid residues are from the NifD subunits.

Table S2. Relevant NifH amino acid residues conserved in NifH^{RS} and their expected roles.

**NifH^{Av} residue***	**NifH^{RS} residue**	**Role**
C97	C95	$[Fe_4S_4]$ binding
C132	C130	
A98	G96	
V130	V128	$[Fe_4S_4]$ interaction
F135	F133	
R100	R98	
E112	L110	
R140	R138	NifDK protein interaction
K143	Y141	
M58	L57	
V67	-	

* NifH^{Av} amino acid residue numbering without the N-terminal methionine.

Table S3. Activity of $NifH^{Av}$ and $NifH^{RS}$ in nitrogenase catalysis (C_2H_2 and N_2 reduction), FeMo-co synthesis, and P-cluster formation.

**NifH activity (Substrate)**	**nmol (C_2H_4 or NH_3)$\cdot min^{-1}\cdot mg^{-1}$ $NifDK^{Av}$**	
	**$NifH^{Av}$**	**$NifH^{RS}$**
Reductase (C_2H_2)	1926 ± 94	301 ± 32
Reductase (N_2)	670 ± 25	141 ± 19
FeMo-co synthesis (C_2H_2)	715 ± 47	74 ± 29
P-cluster formation (C_2H_2)	243 ± 62	29 ± 4

Reductase activities were determined as described in Supporting Information Methods ($NifH^{RS}$ dinitrogenase reductase activity). Activities in FeMo-co synthesis were estimated using P-cluster containing apo-$NifDK^{Av}$ (see $NifH^{RS}$ FeMo-co synthesis activity in SI Methods). Control reactions of nitrogenase reconstitution by the simple addition of isolated FeMo-co (FeMo-co insertion assays) yielded 521 ± 117 nmol $C_2H_4\cdot min^{-1}\cdot mg^{-1}$ $NifDK^{Av}$. P-cluster formation activities were estimated in a three-step assay (see $NifH^{RS}$ P-cluster maturation activity in SI Methods): incubation of $NifH^{Av}$ (or $NifH^{RS}$) with apo-$NifDK^{Av}$ for P-cluster maturation, addition of pure FeMo-co for its insertion into apo-$NifDK^{Av}$, and determination of the acetylene reduction activity of reconstituted $NifDK^{Av}$. Negative control reactions lacking the first step but using $NifH^{Av}$ or $NifH^{Rs}$ for the acetylene reduction step yielded 27 ± 10 or 5 ± 1 nmol $C_2H_4\cdot min^{-1}\cdot mg^{-1}$ $NifDK^{Av}$, respectively. Reactions using $NifH^{Rs}$ in the P-cluster maturation step and $NifH^{Av}$ in the acetylene reduction yielded 155 ± 27 nmol $C_2H_4\cdot min^{-1}\cdot mg^{-1}$ $NifDK^{Av}$. The substrate used for each activity is indicated in parenthesis. Data are average activities ± SD ($n \geq 2$).

Table S4. Conserved amino acid residues expected to be involved in RS, K1 and K2-cluster binding in NifB^{RS} by comparison with *M. thermoacetophila* NifB (NifB^{Mt}).

**NifB^{Mt} residue**	**NifB^{RS} residue**	**Cluster bound (Motif)**
C49	C44	RS-cluster (Cx$_3$Cx$_2$C motif)
C53	C48	
C56	C51	
C62*	C57	
C29	C24	K1-cluster (HPC and ExRP motifs)
C128	C123	
H42	H37	
E65	E60	
C273	C268	K2-cluster (Cx$_2$CRxDA motif)
C276	C271	
D279	D274	
H27	H22	HPC motif

*C62 residue is not always a ligand of the RS-cluster as it is displaced by SAM to initiate catalysis.(13)

Table S5. Inhibitory effect of W on apo-NifDK^{Av} and apo-NifDK^{RS} reconstitution.

[WO_4^{2-}] µM	Apo-NifDK^{Av} Mo added first * (% activity)	Apo-NifDK^{RS} Mo added first* (% activity)	Apo-NifDK^{RS} W added first** (% activity)
0 (x 0)	100.0	100.0	100.0
17.5 (x 1)	98.5	94.6	100.9
175 (x10)	97.7	97.1	96.1
350 (x 20)	94.5	83.9	104.7
875 (x 50)	83.2	78.5	84.5
1750 (x 100)	63.2	68.1	69.4

* In these assays, apo-NifDK was incubated with molybdate before tungstate addition.

** In these assays, apo-NifDK^{RS} was incubated with tungstate before molybdate addition.

The number in parenthesis indicate the excess of tungstate concentration with respect to molybdate (17.5 µM) in the assay. Reactions with 17.5 µM molybdate and no tungstate yielded 389.4 ± 76.9 $C_2H_4\cdot min^{-1}\cdot mg^{-1}$ NifDK^{Av} and 115.06 ± 32.15 $C_2H_4\cdot min^{-1}\cdot mg^{-1}$ NifDK^{RS} (100% activity). Reactions without molybdate and 100 µM tungstate yielded 43.2 ± 8. $C_2H_4\cdot min^{-1}\cdot mg^{-1}$ NifDK^{Av} (11% activity) and 14.2 $C_2H_4\cdot min^{-1}\cdot$mg-^{1} NifDK^{RS} (12% activity). Reactions with neither molybdate nor tungstate yielded 44.4 ± 3.1 $C_2H_4\cdot min^{-1}\cdot mg^{-1}$ NifDK^{Av} (11% activity) and 15.5 ± 0.6 $C_2H_4\cdot min^{-1}\cdot$mg-^{1} NifDK^{RS} (13% activity). Data are average ± SD of at least 2 independent reactions.

Table S6. Plasmids used in this work.

Plasmid	DNA Construct	Source
pDB6	*nifHDK*^{Av} region	Dennis Dean
pDB2080	Δ*vnfH*::Km^{R}	Dennis Dean
pASC36	pDB6-*nifH*^{RS}-*nifDK*^{Av}	This study
pRHB513	pET16b-his-*nifH-nifBDK*^{RS}	This study
pN2LP123	pET16b-TwinStreptagII-TEV-*nifB*^{RS}	This study
pN2LP49	pTRC99a-*StrepTagII-nifDK*^{RS}	This study
pETDuet-1	pRSF-*isc-metK*-Duet-1	J. Fontecilla / Y. Nicolet
pRHB608	pGEMT-*nifUS*	L. Rubio
pN2LP30	pRSF-*nifUS*^{Av}-*metK*-Duet-1	This study
pN2LP50	pRSF-*isc-nifH*^{RS}Duet-1	This study
pN2LP51	pRSF-*nifUS*^{Av}-*nifH*^{RS}Duet-1	This study
pN2SB73	Km^{r} pTRC-99a derivative	L. Rubio
pN2LP41	pN2SB73- *his-nifB*^{RS}	This study
